# Lactate‐induced metabolic reprogramming of TAMs impairs antigen presentation capacity via C/EBPα–CD74 axis in oral squamous cell carcinoma

**DOI:** 10.1002/ctm2.70639

**Published:** 2026-03-10

**Authors:** Mengyao Wang, Mengqi Wang, Zizhen Gong, Fanrui Zeng, Zihui Ni, Rundong Zhai, Weiwen Zhu, Jiayi Zhang, Laikui Liu

**Affiliations:** ^1^ Department of Basic Science of Stomatology The Affiliated Stomatological Hospital of Nanjing Medical University Nanjing China; ^2^ State Key Laboratory Cultivation Base of Research, Prevention and Treatment for Oral Diseases Nanjing Medical University Nanjing China; ^3^ Jiangsu Province Engineering Research Center of Stomatological Translational Medicine Nanjing Medical University Nanjing China; ^4^ Department of Oral Mucosal Diseases The Affiliated Stomatological Hospital of Nanjing Medical University Nanjing China

**Keywords:** antigen presentation, CD74, oral squamous cell carcinoma, tumour‐associated macrophages

## Abstract

**Background:**

Throughout oral squamous cell carcinoma (OSCC) progression, tumor‐associated macrophages (TAMs) lose their antigen‐presenting capacity and anti‐tumor function. The mechanisms that cause this dysfunction are not fully understood. CD74 is essential for antigen‐presenting process, while little direct evidence describes its role in TAMs' immune function.

**Methods:**

We integrated single‐cell transcriptomic analysis, clinical cohort validation and CD74 conditional knockout mouse model to investigate the role of CD74 in TAMs during OSCC progression. Metabolomic analysis and mechanistic studies were performed to dissect how lactate‐mediated metabolic reprogramming regulates CD74 expression.

**Results:**

We demonstrate that lactate accumulation in TME induces metabolic reprogramming of TAMs, which drives the acetylation of C/EBPα, and consequently suppresses CD74 expression. This downregulation of CD74 impairs the antigen‐presenting capacity of TAMs, suppresses T cell activation, and ultimately promotes OSCC progression and recurrence.

**Conclusions:**

Our findings reveal the critical role of the lactate‐C/EBPα‐CD74 axis in shaping TAMs function, and provide potential therapeutic target for OSCC immunotherapy.

**Key points:**

- CD74^hi^ TAMs decrease during OSCCprogression and are associated with patient prognosis.
- Loss of CD74 impairs antigen presentation andsuppresses T cell activation.
- Tumor‐derived lactateinhibits CD74 transcription via C/EBPα acetylation.

## Graphical abstract

• CD74^hi^ TAMs correlate with favourable prognosis in OSCC.

• Lactate reprograms TAMs metabolism and increases mitochondrial respiration.

• C/EBPα acetylation suppresses CD74 and impairs antigen presentation.

• Restoring CD74 enhances T‐cell activation and limits OSCC progression.

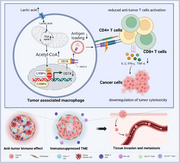

## 1 INTRODUCTION

Oral squamous cell carcinoma (OSCC) is the most common type of head and neck squamous cell carcinoma (HNSCC) which ranks as the sixth‐most common cancer worldwide. Postoperative recurrence and lymphoid node metastasis remain key factors affecting the prognosis of OSCC patients.^1^, ^2^ Since the advent of precision medicine and the application of immune checkpoint blockade (ICB) therapies,^3^, ^4^ the tumour microenvironment (TME) has been recognised as a key factor that influences tumour progression and prognosis.^5^ Tumour‐associated macrophages (TAMs) are considered as the most abundant immune cell type in TME.^6^ TAMs play critical roles in regulating anti‐tumour immunity due to their remarkable functional plasticity and heterogeneity.^6^, ^7^ On the one hand, TAMs could promote the differentiation of regulatory T cells (Treg) while inhibit tumour cell killing activity of CD8^+^ T cell via PD‐L1/PD‐1, thereby suppressing T‐cell‐mediated anti‐tumour immunity.^8^ On the other hand, they could adopt a pro‐inflammatory phenotype, with increased TNF‐α and IL‐12 secretion, natural killer (NK) cell activation, direct tumour cell phagocytosis and T‐cell cytotoxicity stimulation via antigen presentation.^9^ The molecular mechanisms governing the phenotype plasticity of TAMs have not been fully understood yet.

As one of the potential anti‐tumour functions of TAMs, antigen presentation determines both the extent and quality of T‐cell activation, laying the foundation for anti‐tumour immune responses.^10^, ^11^, ^12^ Effective anti‐tumour responses require the coordinated activation of both CD8^+^ cytotoxic T lymphocytes (CTLs) and CD4^+^ helper T cells.^13^ Among them, the activation of CD4^+^ T cells during tumour progress which requires MHC class II‐restricted antigen recognition, is primarily dependent on local antigen presentation within the TME.^14^, ^15^ Among all the professional antigen‐presenting cells (APCs), TAMs are the most abundant cell type who accumulate in TME, act uniquely in adaptive immunity.^16^ Consequently, TAMs‐mediated MHC II antigen presentation would be indispensable in initiating and sustaining anti‐tumour immunity. Through tumour cell phagocytosis, TAMs degrade proteins into peptide fragments within lysosomes. TAMs load those peptide onto MHC II molecules and then present the antigens at the cell surface for recognition by T‐cell receptors (TCRs), thus initiating downstream immune responses.^15^, ^17^ Notably, the antigen‐presenting capacity of TAMs is often impaired during tumour progression, causing T‐cell exhaustion and immune evasion.^10^ The mechanisms of dynamic regulation during antigen presentation by TAMs remain unclear.

CD74, a non‐polymorphic type II transmembrane protein that serves as an invariant chain of MHC II, is required for MHC II complex assembly, trafficking and peptide loading.^18^ Recent research has revealed that CD74 can restore MHC II‐associated antigen presentation in B cell lymphomas with IRF8 mutations, thus suppressing immune escape.^19^ Additionally, in solid tumours, CD74^+^ stromal cell abundance within the TME correlated with improved responsiveness to immunotherapy.^20^ However, there is limited documentation on the specific roles of CD74 in TAMs, and direct evidence of CD74 in their immune function is rarely reported. Therefore, unveiling functional characterisation and regulatory mechanisms of CD74 in TAMs is crucial for providing critical insights into the restoration of macrophage antigen presentation.

Metabolic reprogramming, a hallmark of both cancer and immune regulation, serves as a critical interface between environmental cues and cellular function.^21^ Tumour cells often utilise aerobic glycolysis to sustain rapid proliferation, increasing glucose uptake and lactate production, even in the presence of oxygen—a phenomenon known as the Warburg effect.^22^ This metabolic shift creates a TME with hypoxia and high lactate concentrations. As a metabolically flexible cell population, TAMs can take up and respond to environmental metabolites such as lactate, potentially reshaping their transcriptional and functional identity.^23^ In our research, we found clues by single‐cell RNA sequencing (scRNA‐seq) that metabolic reprograming regulate TAMs antigen presentation capacity, and proved the key role of lactate. Monocarboxylate transporter 1 (MCT1) facilitates lactate importation into TAMs, where it is utilised as a carbon source to fuel the tricarboxylic acid (TCA) cycle within mitochondria and modulate gene expression and cell fate.^23^, ^24^ Additionally, acetyl‐CoA, a critical TCA intermediate, serves as a substrate for the acetylation of both histone and non‐histone proteins, thus contributing to the epigenetic regulation of gene expression.^25^, ^26^, ^27^ Although lactate‐induced histone acetylation could suppress inflammatory gene programs in macrophages,^28^ the precise molecular pathways through which lactate regulates antigen presentation remain unclear.

Here, we explore the role of CD74^hi^ TAMs as APCs within the OSCC microenvironment, focusing on how metabolic signals disrupt their antigen presentation capacity. According to the results, there are decreased CD74^hi^ TAMs during the progression of OSCC. TAMs took up lactate that accumulated within the TME, leading to overactivation of oxidative phosphorylation (OXPHOS), which, in turn, promoted the acetylation of C/EBPα, the key transcription factor (TF) of CD74. As a result, TAMs exhibited reduced CD74 transcription and compromised MHC II‐mediated antigen presentation. These findings highlight a novel immunometabolic axis that links tumour‐derived lactate with antigen presentation capacity in TAMs.

## 2 EXPERIMENTAL SECTION/METHODS

### 2.1 Clinical samples

Histological staining was performed on formalin‐fixed paraffin‐embedded (FFPE) specimens obtained from 61 OSCC patients between January 2013 and December 2015 at the Affiliated Stomatological Hospital of Nanjing Medical University. Additionally, scRNA‐seq was performed on fresh tumour tissues from four OSCC patients treated between January 2023 and December 2023.

### 2.2 Single‐cell sequencing

After preparing the sample into a highly viable single‐nucleus suspension, gel beads‐in‐emulsions (GEMs) are generated using the 10× genomics microfluidic platform to enable reverse transcription and cDNA synthesis within each individual nucleus incorporating a specific barcode. Following emulsion breakage, the cDNA is amplified via PCR and subjected to quality control. The cDNA is then fragmented, end‐repaired, ligated with adapters and indexed to construct a sequencing library. Finally, sequencing is performed on the Illumina NovaSeq platform to obtain single‐nucleus transcriptome data.

### 2.3 Single‐cell RNA sequencing data processing, cluster annotation and data integration

The filtered matrices were converted into sparse matrices using the Seurat package (v4.4.0) with parameters set to min.cells = 3 and min.features = 40 (i.e., genes expressed in at least three cells and cells expressing at least 40 genes). Quality control was performed based on the following criteria: nFeature_RNA > 300 and nFeature_RNA < 4000, percent.mt < 15, and nCount_RNA > 1000 (i.e., only cells with 300–4000 detected genes, mitochondrial gene expression below 15% and a total RNA count exceeding 1000 were retained). Additional cell filtering was conducted using the doubletFinder_v3 method from the DoubletFinder package (v2.0.3) to remove potential doublets. The filtered data were normalised using the NormalizeData function in Seurat to eliminate technical variation. Highly variable genes were identified via the FindVariableFeatures function, as these genes are more informative for downstream analysis. Data were subsequently scaled using the ScaleData function, followed by principal component analysis (PCA) using the RunPCA function. The ‘RunUMAP’ function was then used to reduce the dimensionality of the data. Data integration was executed post‐normalisation and scaling, with batch effect correction performed using the RunHarmony function from the Harmony package (v1.1.0). Following correction, neighbourhood identification was conducted using the FindNeighbors function (dim = 20), and clustering analysis was performed via the FindClusters function, with resolutions set to 1 for major cell types and  .6 for sub‐clusters. Dimensionality reduction and visualisation were carried out using the RunUMAP function. Cell clusters were annotated based on the expression of specific feature genes. The normalisation and processing steps for extracted sub‐populations were identical to the aforementioned workflow.

### 2.4 Acquisition and processing of public scRNA‐seq data

Two independent OSCC transcriptomic datasets were retrieved from the Gene Expression Omnibus (GEO) database (https://www.ncbi.nlm.nih.gov/geo/) for external validation:

GSE41613: Contributed by Lohavanichbutr et al., this dataset comprises 167 samples (97 OSCC tissues and 70 control tissues) analysed via the Affymetrix HG‐U133 Plus 2.0 platform. Detailed survival follow‐up data were provided for this cohort.

GSE65858: Contributed by Wichmann et al., this dataset includes 270 primary OSCC samples analysed using the Illumina HumanHT‐12 V4.0 platform, also accompanied by comprehensive survival metadata.

### 2.5 CIBERSORTx analysis

A biologically specific reference signature was constructed using internal scRNA‐seq myeloid cell data: Signature Construction: Candidate markers were identified from the top 20 marker genes of each myeloid sub‐cluster. To ensure sub‐cluster specificity, a stringent filtering criterion of an adjusted *p* value (*p*_adj) < .01 and a percentage difference (*d* = pct.1 − pct.2) > .2 was applied. Deconvolution: Using this custom reference signature, the cleaned bulk gene expression matrices from GSE41613 and GSE65858 were uploaded to the CIBERSORTx platform (https://cibersortx.stanford.edu/) for immune cell deconvolution.

### 2.6 Multiplex immunofluorescence histochemical staining

For multiplex immunofluorescence histochemical (mIHC) staining tissue sections were dewaxed in xylene and rehydrated through a graded ethanol series, followed by three 3‐min washes with PBS buffer. Antigen retrieval was achieved through heat‐induced epitope retrieval using citrate buffer. After the incubation with antibodies and TSA signal amplification staining solution, repeated microwave‐assisted antigen retrieval steps were required. After the final labelling, nuclear counterstaining was performed using DAPI.

To evaluate the results of mIHC for CD74^hi^ TAMs, the whole slides were scanned. Subsequently, five high‐magnification fields (400×) with the highest CD68^+^ cell density were selected per section, and CD74^+^CD68^+^ double‐positive and CD68 single‐positive cells were quantified, with their ratios averaged across fields. The average of the five ratios was used as the final evaluation of the infiltration proportion of CD74^hi^ TAMs. Two experienced pathologists independently performed the evaluation, with discrepancies resolved through consensus.

### 2.7 Animal models

To induce CD74 deletion before tumour implantation, conditional CD74 knockout (*Cd74^fl/fl^; Lyz2‐Cre^ERT2^*) and control (*Cd74^fl/fl^*) mice aged 5–8 weeks were administered tamoxifen.

For the subcutaneous OSCC model, MTCQ1 OSCC cells (5 × 10^5^) were resuspended in in 100 µL DMEM and injected subcutaneously into the right flank of the mice. Tumour volume was measured every 3 days (*V* = .5 × *L* × *W* ^2^), and the mice were sacrificed at tumour volume >1500 mm^3^ or weight loss >20%.

For the OSCC tongue injection model, MTCQ1 cells (5 × 10^5^ in 50 µL) were injected into the tongue mucosa, and a soft diet was provided post‐injection. Tumour volume was measured every 3 days (*V* = .5 × *L* × *W* ^2^), and the mice were sacrificed 30 days after injection.

For the recurrence model of subcutaneously implanted tumour, established subcutaneous tumours were surgically resected 30 days after the subcutaneous MTCQ1 injection. A residual tumour tissue with the diameter of  .5 mm was placed in the surgical area before suture, and the mice were sacrificed 14 days after injection.

For the comparison of TAMs between primary and recurrent tumour, subcutaneous tumours were implanted in the left flank of the mice on the 14th day after the first tumour model establishment. The recurrence model was established as described, and tumour tissues from both flanks were collected after 14 days of the surgery.

### 2.8 Antigen presentation assays

Macrophages were starved for 24 h and then pulsed with OVA_323_–_339_ peptide (1 µg/mL) for 48 h. CFSE‐labelled OT‐II CD4^+^ T cells were co‐cultured with macrophages at a 5:1 ratio, after which T‐cell proliferation and activation were evaluated after 3–5 days by flow cytometry.

### 2.9 Cell Mito Stress Test and Glycolytic Rate Assay

Cells are seeded into Seahorse XF culture plates. After adherence, the medium is replaced with Seahorse XF DMEM medium for pre‐incubation for 1 h. The Seahorse XF culture plates are loaded on the Agilent Seahorse XFe/XF Analyzer, and the basal ECAR and OCR are recorded. For Cell Mito Stress Test, the cells are treated with a sequential injection of 1 µM oligomycin,  .5 µM FCCP and 1 µM rotenone/antimycin A. For Glycolytic Rate Assay, rotenone/antimycin A and glycolysis inhibitor 2‐DG are injected sequentially.

### 2.10 Co‐immunoprecipitation

Cells are treated with lysate, and incubated with the target antibody at 4°C overnight, and then Protein A/G magnetic beads are added for rotary incubation overnight. Magnetic beads are adsorbed using a magnetic rack; after washing, 1× loading buffer is added, and the mixture is heated at 95°C for 10 min to denature and release proteins. The magnetic beads are removed, and the obtained proteins are used for subsequent Western blot (WB) detection.

### 2.11 CUT & RUN

The CUT&RUN assay was performed using the CUT&RUN kit (Vazyme). Briefly, 1 × 10^6^ cells were resuspended in permeabilisation buffer containing  .1% digitonin. Target antibodies and magnetic beads were added, followed by static incubation at 4°C for 1 h. The supernatant was then discarded, and pA‐MNase complex was added with shaking and mixing at 4°C for 30 min. Then, CaCl_2_ was added to initiate enzymatic cleavage. The supernatant was transferred to a stop solution which containing EDTA and incubated at 65°C for 2 h to reverse cross‐linking. After that, DNA can be purified from the samples and analysed by qPCR.

### 2.12 Metabolomic analysis

For metabolomics detection, cells are frozen in liquid nitrogen and then mixed with 80% methanol. After centrifugation, the supernatant is collected and concentrated to dryness. The samples are reconstituted before being analysed instrumentally for metabolites. After peak extraction, alignment and normalisation of the raw data, pathway enrichment analysis and differential metabolite analysis are conducted.

### 2.13 Statistical analysis

Data analysis was performed using GraphPad Prism 8.0. Two groups were compared using Student's *t*‐test while multiple groups were analysed by the one‐way ANOVA. Correlation analysis was conducted using the Spearman's rank correlation. A *p* value <.05 was considered statistically significant. Significance levels were denoted as: *p < .05; **p < .01; ns = not significant.

## 3 RESULTS

### 3.1 CD74^hi^ TAMs infiltration correlates with prognosis in OSCC patients

**FIGURE 1:**
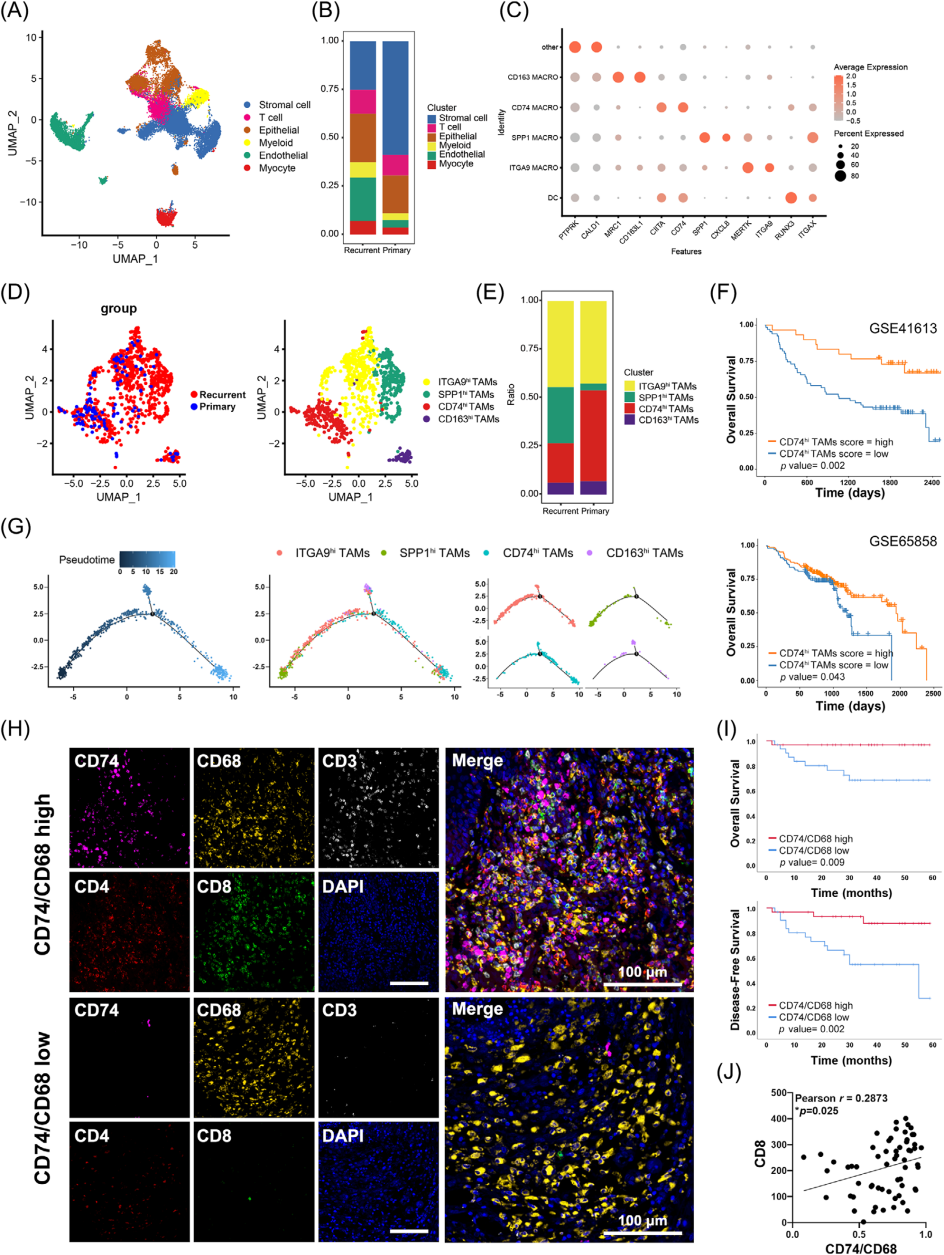
CD74^hi^ TAMs infiltration correlates with prognosis in OSCC patients. (A) The t‐SNE plot of the single‐cell RNA‐seq analysis of OSCC samples (*n* = 4) shows that six major clusters annotated by representative marker genes. (B) Proportions of different clusters in recurrent tumours (*n* = 3) and primary tumour (*n* = 1). (C) Bubble plot highlights the marker genes for myeloid sub‐populations. (D) UMAP plot shows the patients group (left) and the distribution of TAMs sub‐clusters (right). (E) The proportions of TAMs sub‐clusters shows decreased proportion of CD74^hi^ TAMs in recurrent tumours. (F) Patients with high CD74^hi^ TAMs score showed better survival in GSE41613 (upper, *p* = .0016) and GSE65858 (lower, *p* = .043) cohorts. (G) Pseudotime trajectory analysis of tumour‐infiltrating macrophages and differentiation states of distinct TAMs sub‐populations show that CD74^hi^ TAMs could represent a terminally differentiated sub‐population; each dot represents a cluster and branches indicate differentiation states. (H) Multiplex immunohistochemistry images for CD74, CD68, CD3, CD4 and CD8 in OSCC tissues with high (top) and low (bottom) CD74/CD68 score Scale bar: 100 µm. (I) Patients with high CD74/CD68 scores have better overall survival (upper, *p* = .009) and disease‐free survival (lower, *p* = .002). (J) Correlation analysis between CD8^+^ T‐cell counts and CD74/CD68 scores (*r* = .2873, *p* = .025).

To evaluate the phenotype of TAMs in OSCC, fresh tumour tissues from four OSCC patients were subjected to scRNA‐seq. After quality control and normalisation, PCA and clustering were performed, yielding six major cell types, including stromal cells, T cells, epithelial cells, myeloid cells, endothelial cells and myocytes, as visualised with t‐SNE projection (Figures 1A and S1A). Annotation of clinical origin revealed that recurrent tumours exhibited a marked stromal cell downregulation and endothelial and myeloid cell upregulation (Figure 1B). As the most abundant population of immune cells, myeloid cells were further extracted and re‐clustered into eight subtypes (Figure S1B,C). Among them, four transcriptionally distinct sub‐populations of TAMs were identified: CD163^hi^ TAMs, CD74^hi^ TAMs, SPP1^hi^ TAMs and ITGA9^hi^ TAMs (Figure 1C). Representative marker genes for CD74^hi^ TAMs included CD74, CIITA and HLA‐DRA, which were associated with antigen processing and presentation decreased their expression in recurrent tumours (Figure S1D). These findings collectively suggest that CD74^hi^ TAMs possess the potential for robust MHC class II‐mediated antigen presentation.

Primary and recurrent tumours showed no significant difference in the proportions of ITGA9^hi^ and CD163^hi^ TAMs, while the latter exhibited significantly lower CD74^hi^ TAMs proportions (Figure 1D,E), implying the loss of antigen presentation function within the TAMs compartments. Pseudotime analysis further revealed that CD74^hi^ TAMs could represent a terminally differentiated sub‐population with potential lineage conversion from other TAMs subsets (Figure 1G). We derived the markers of CD74^hi^ TAMs and evaluated CD74^hi^ TAMs scores of RNA‐seq datasets GSE41613 and GSE65858 by deconvolution. The results revealed that patients with higher scores had better survival rate (Figure 1F).

To further evaluate the clinical relevance of CD74^hi^ TAMs, formalin‐fixed tissue sections from 61 OSCC patients were subjected to mIHC staining, with CD74 and CD68 co‐staining revealing varying degrees of CD74^hi^ TAMs infiltration across patients (Figures 1H and S2A). Notably, patients with higher CD74 expression in TAMs exhibited markedly increased infiltration of CD4^+^ and CD8^+^ T cells, suggesting that CD74^hi^ TAMs are linked to an immune‐inflamed TME. Additionally, CD74 expression in the TAMs was quantified using the ratio of CD74^hi^CD68^+^ to total CD68^+^ TAMs (CD74/CD68 score). According to the cross‐analysis results, the CD74/CD68 score correlated significantly with tumour differentiation grade, lymph node metastasis (LNM), clinical stage and recurrence status, but not with tumour size (Table 1).

**TABLE 1:** Correlation of CD74/CD68 score and clinical information of OSCC patients.

	CD74/CD68 score				
	Low		High		
	Count	Percent	Count	Percent	*p* value
Gender					
Female	13	41.90%	13	43.30%	.912
Male	18	58.10%	17	56.70%	
Age					
<62	14	45.20%	15	50.00%	.705
≥70	17	54.80%	15	50.00%	
Location					
Buccal	11	35.50%	11	36.70%	.916
Tongue	7	22.60%	6	20.00%	
Gingival	3	9.70%	5	16.70%	
Oral floor	2	6.50%	1	3.30%	
Others	8	25.80%	7	23.30%	
Pathologic differentiation					
I	15	48.40%	25	83.30%	.004^**^
II/III	16	51.60%	5	16.70%	
Invasive pattern					
WPOI: 1–3	11	35.50%	14	46.70%	.375
WPOI: 4–5	20	64.50%	16	53.30%	
Tumour size					
T1/T2	22	71.00%	24	80.00%	.413
T3/T4	9	29.00%	6	20.00%	
Lymph node metastasis					
N0	16	51.60%	25	83.30%	.008^**^
N1/N2	15	48.40%	5	16.70%	
Clinical stage					
I/II	13	41.90%	21	70.00%	.027^*^
III/IV	18	58.10%	9	30.00%	
Recurrence					
No	17	54.80%	27	90.00%	.002^**^
Yes	14	45.20%	3	10.00%	

*Note*: Association of CD74/CD68 score with gender, age, tumour location, pathological differentiation, invasive pattern, tumour size, LNM, clinical stage and recurrence status.

*p<.05, **p<0.

According to Kaplan–Meier (K–M) survival analysis, patients with high CD74^hi^ TAMs infiltration exhibited significantly improved overall survival (OS) and disease‐free survival (DFS; both *p* < .01, Figure 1I). Analysis between clinical issues and CD74/CD68 score revealed no significant differences across different invasion pattern groups (*p* = .0624), while significant associations were found with clinical stage (*p* = .026), pathologic stage (*p* < .001) and LNM (*p* = .019; Figure S2B). Furthermore, immunostaining for CD8^+^ T cells revealed that the CD74/CD68 score correlated positively with CD8^+^ T‐cell infiltration (*r* = .2873, *p* = .025, Figures 1J and S1E), suggesting the involvement of CD74^hi^ TAMs in T‐cell recruitment or activation.

### 3.2 CD74 of TAMs governs antigen presentation capacity

**FIGURE 2:**
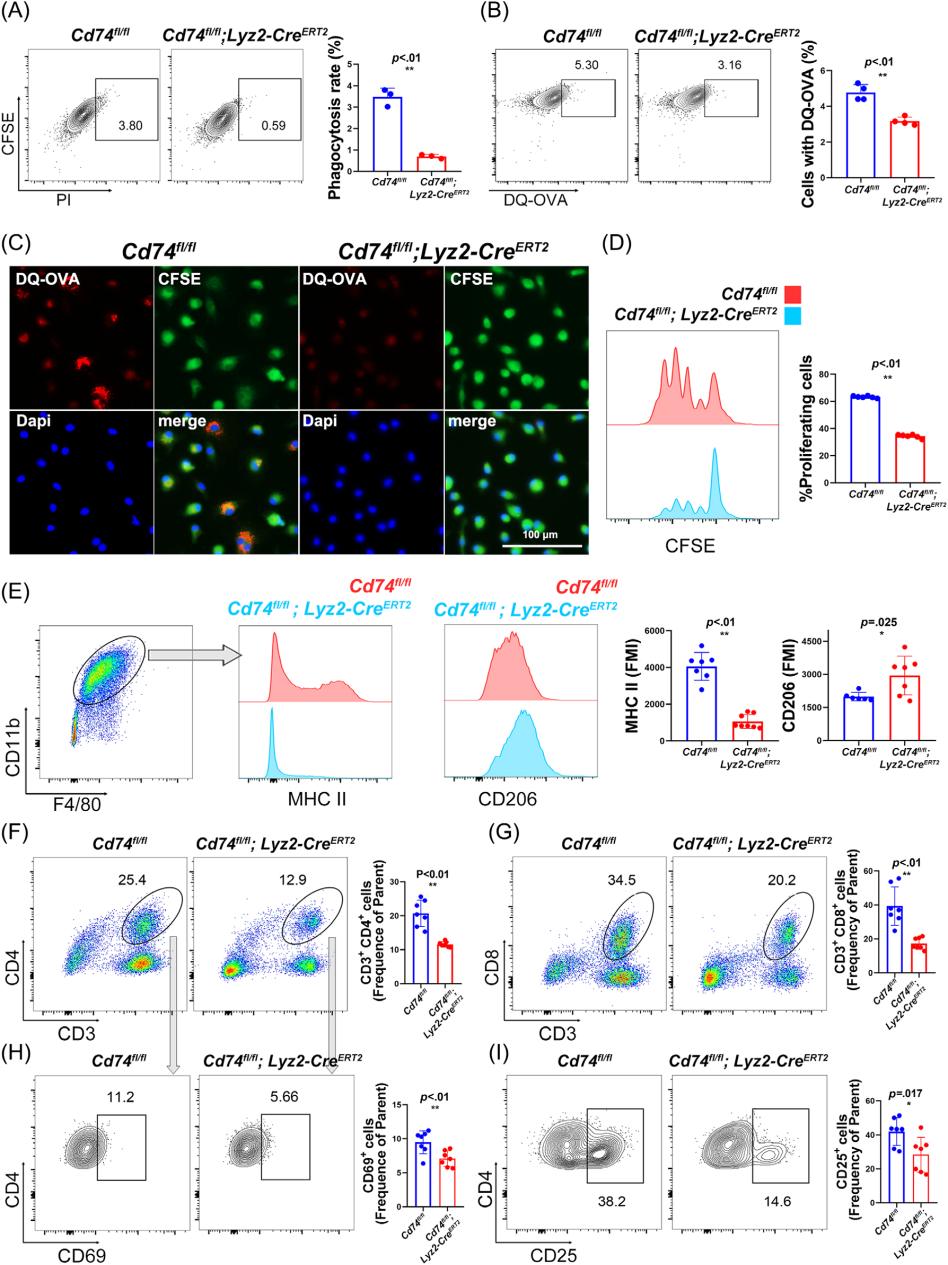
CD74 of TAMs governs antigen presentation capacity and regulates T cells activation. (A) Comparison of phagocytosis capacity between BMDMs from *Cd74^fl/fl^; Lyz2‐Cre^ERT2^* and *Cd74^fl/fl^* mice (*p* < .01). (B) Comparison of antigen processing capacity between BMDMs from *Cd74^fl/fl^; Lyz2‐Cre^ERT2^* and *Cd74^fl/fl^* mice (*p* < .01). (C) Representative fluorescence images showed antigen processing by TAMs from *Cd74^fl/fl^; Lyz2‐Cre^ERT2^* mice versus *Cd74^fl/fl^* mice. Scale bar: 100 µm. (D) CD74 knockout significantly reduced exogenous antigen presentation to CD4^+^ T cells, with fewer proliferating T cells (*p* < .01). (E) Myeloid‐specific CD74 deletion reduces MHC II expression and increases CD206 expression in TAMs (*p* < .01 and *p* = .025, respectively). (F, G) Flow cytometry shows significantly reduced infiltration of CD3^+^CD4^+^ and CD3^+^CD8^+^ T cells in recurrent tumour TILs (both *p* < .01). (H, I) The expression of CD69 and CD25 on intratumoural CD4^+^ T cells are significantly downregulated in *Cd74^fl/fl^; Lyz2‐Cre^ERT2^* groups (*p* < .01 and *p* = .017, respectively).

Since there is no direct evidence describes CD74 functions in TAMs, we generated myeloid‐specific CD74 conditional knockout (CKO) mice (*Cd74^fl/fl^; Lyz2‐Cre^ERT2^*) via tamoxifen treatment to investigate the impact of CD74 knockdown on TAMs phagocytosis, antigen presentation, and T cells activation. We first examined antigen processing using DQ‐OVA, a self‐quenched fluorescent antigen. Extensive intracellular co‐localisation of processed DQ‐OVA fragments with the CD74 protein was observed in TAMs isolated from murine OSCC tumours (Figure S3), indicating CD74 involvement in antigen processing within macrophages. Then, subcutaneous OSCC tumours were induced in *Cd74^fl/fl^; Lyz2‐Cre^ERT2^ and Cd74^fl/fl^* mice, with TAMs later harvested from the tumours and examined for antigen presentation function. According to the phagocytosis assays, CD74‐deficient BMDMs exhibited a significantly reduced phagocytic capacity (*p* < .01, Figure 2A). Additionally, DQ‐OVA‐based antigen processing assays revealed reduced fluorescence intensity in both BMDMs and TAMs without CD74, indicating impaired antigen degradation (*p* < .01, Figure 2B,C).

To assess MHC class II‐dependent antigen presentation, OVA_323–339 peptide‐pulsed CD74‐deficient TAMs were co‐cultured with OT‐II CD4^+^ T cells. The CD74‐deficient group exhibited significantly diminished T‐cell proliferation and activation (Figure 2D), confirming the essential role of CD74 in antigen uptake, processing and presentation by TAMs. Flow cytometry of TAMs from *Cd74^fl/fl^; Lyz2‐Cre^ERT2^* mice further revealed MHC class II downregulation and CD206^+^ immunosuppressive macrophage upregulation (Figure 2E), implying that CD74 deletion skewed TAMs polarisation towards an immunosuppressive phenotype. Moreover, splenic CD4^+^ and CD8^+^ T cells from tumour‐bearing *Cd74^fl/fl^; Lyz2‐Cre^ERT2^* mice showed an increased expression of the exhaustion marker PD‐1 and decreased IFN‐γ production (*p* < .01, Figure S4), indicating that CD74 loss in myeloid cells could compromise systemic T‐cell immunity.

Flow cytometry analysis of tumour‐infiltrating lymphocytes (TILs) revealed that CD74 deletion significantly reduced CD4^+^ and CD8^+^ T‐cell proportions in CD45^+^ immune cells (both *p* < .01, Figure 2F,G). Moreover, CD4^+^ TILs in *Cd74^fl/fl^; Lyz2‐Cre^ERT^* mice exhibited a reduced expression of activation markers CD25 and CD69 (*p* < .01 and *p* = .017, respectively, Figure 2H,I), highlighting impaired helper T‐cell priming, potentially due to compromised antigen presentation.

### 3.3 Tumour progression and recurrence exacerbated CD74‐dependent antigen presentation dysfunction in TAMs

**FIGURE 3:**
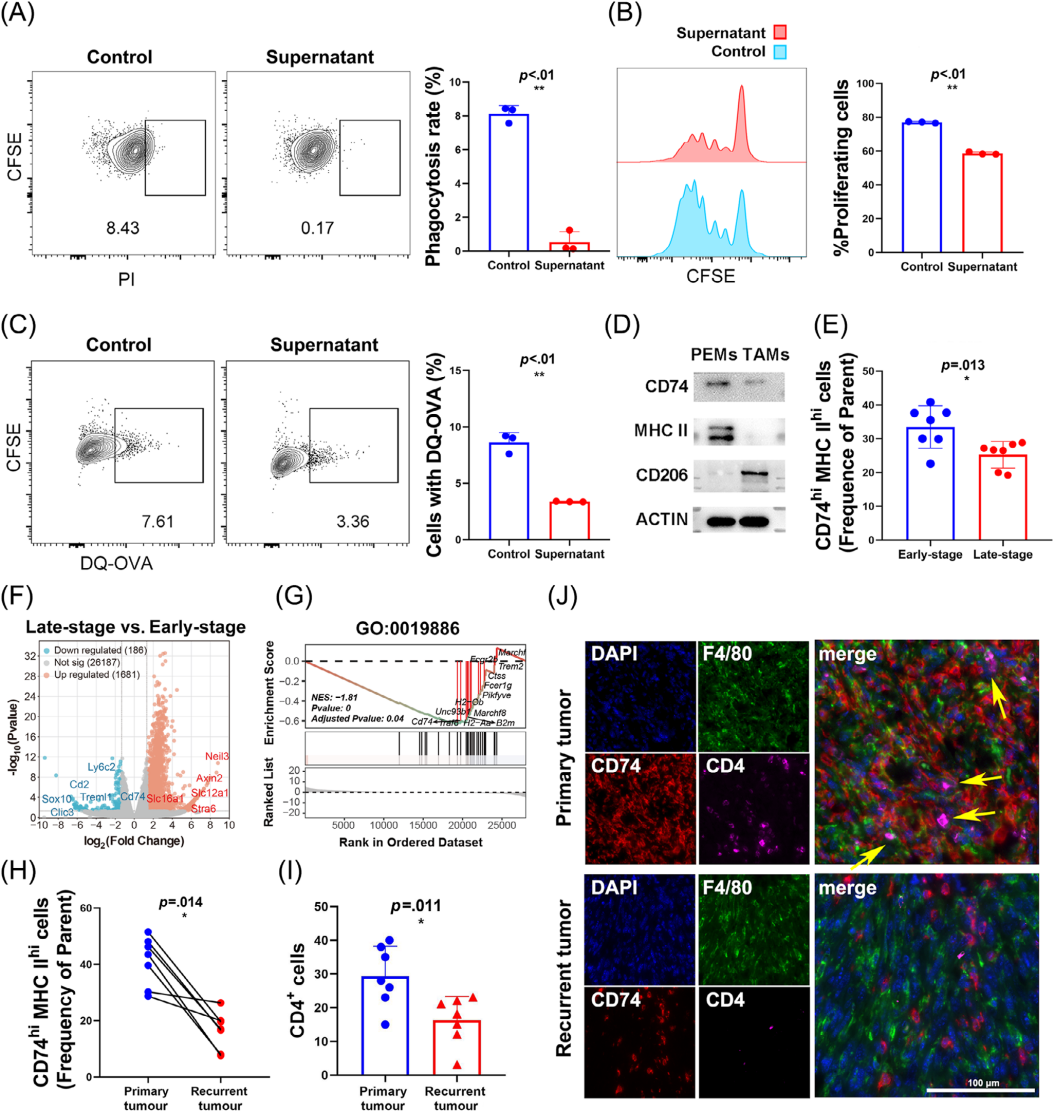
Tumour progression exacerbates CD74‐dependent antigen presentation dysfunction in TAMs. (A) Phagocytosis assay shows that the cell culture supernatant of MTCQ1 significantly reduces BMDM phagocytosis of apoptotic tumour cells (*p* < .01). (B) The cell culture supernatant of MTCQ1 suppresses BMDM antigen presentation–induced proliferation of OT‐II CD4^+^ T cells (*p* < .01). (C) The cell culture supernatant of MTCQ1 inhibits antigen peptide processing in BMDMs (*p* < .01). (D) TAMs show significantly lower CD74 and MHC II expression and higher CD206 compared to PEMs. (E) The proportion of CD74^hi^ MHC II^hi^ cells in TAMs decreases significantly from early‐ to late‐stage OSCC in mice (*p* = .013; *n* = 7 each). (F) Volcano plot showing representative differentially expressed genes in late‐stage versus early‐stage TAMs. (G) GSEA reveals significant downregulation of MHC II‐mediated exogenous antigen presentation pathways in late‐stage TAMs (ES = −1.81, *p* = .04). (H) Flow cytometry shows CD74^hi^ MHC II^hi^ TAMs are significantly reduced in recurrent versus primary tumours (*p* = .014). (I, J) CD4^+^ T‐cell infiltration and co‐localisation with TAMs are significantly reduced in recurrent OSCC (*p* = .011). Yellow arrows indicate contact between CD4^+^ T cells and CD74^hi^ TAMs.

Wild‐type (WT) murine BMDMs were treated with MTCQ1 tumour cell‐conditioned media to simulate tumour conditions in vitro and further explore the dynamic changes in CD74‐dependent antigen presentation during OSCC progression. Conditioned BMDMs exhibited significantly suppressed phagocytic ability, antigen processing and antigen‐presenting capacity (*p* < .01, Figures 3A–C and S5A,B), indicating that the TME dampens macrophage immunocompetence.

In vivo, we established subcutaneous OSCC models in mice and compared TAMs with peritoneal macrophages (PEMs) during early tumour stages. According to the Western blotting (WB) results, compared to PEMs, TAMs exhibited a lower CD74 and MHC II expression and higher CD206 expression (Figure 3D). Notably, this trend intensified in TAMs isolated from late‐stage tumours, as flow cytometry analysis of single‐cell suspensions revealed significantly reduced CD74^hi^ TAMs proportions in late‐stage tumours, which confirming progressive antigen‐presenting impairment in tumour progression (*p* = .013, Figures 3E and S5C–E).

Additionally, RNA‐seq of TAMs from early versus late tumours revealed differential expression of key genes, including *Slc16a1* and *Slc12a1* upregulation and *Sox10* and *Ly6c2* downregulation (Figure 3F). Gene set enrichment analysis (GSEA) further revealed significant suppression of exogenous antigen processing and MHC class II presentation pathways (GO:0019886; ES = −1.81, *p* = .04, Figure 3G), aligning with our phenotypic observations.

The recurrent tumour model showed significantly lower CD74 and MHC II expression in recurrent tumour TAMs compared to primary tumour TAMs from the same mouse (*p* = .014, Figures 3H and S5F). Multiplex IF staining further confirmed reduced infiltration and co‐localisation of CD4^+^ T cells with CD74^hi^ TAMs in recurrent tumours (*p* = .03, Figure 3I,J), suggesting that loss of CD74‐dependent antigen presentation by TAMs could impair helper T‐cell activation.

### 3.4 Loss of CD74 in TAMs promotes progression, recurrence and metastasis of OSCC

**FIGURE 4:**
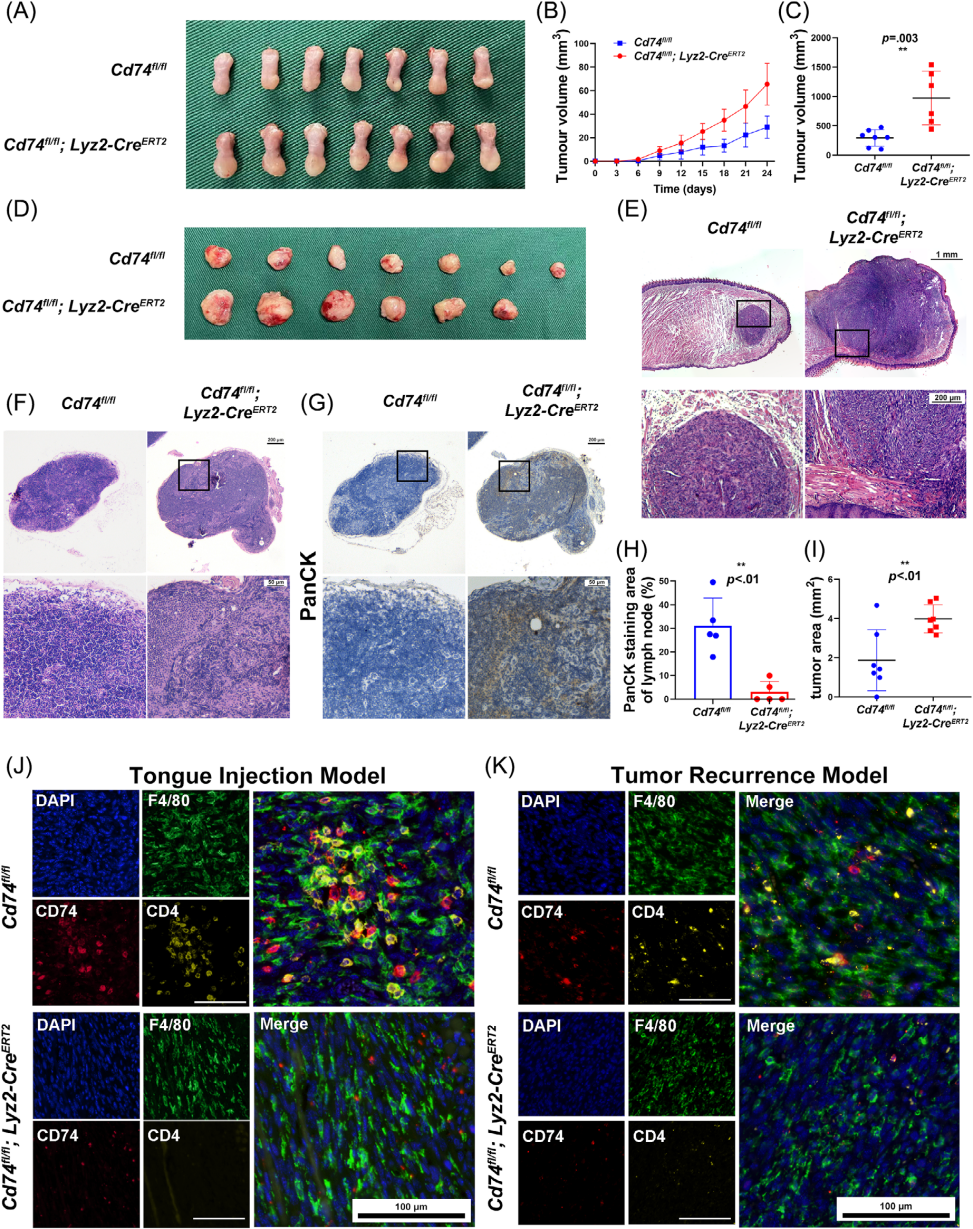
Loss of CD74 in TAMs promotes progression, recurrence and metastasis of OSCC. (A) Representative images of OSCC tongue injection models established in *Cd74^fl/fl^; Lyz2‐Cre^ERT2^* and *Cd74^fl/fl^* mice (*Cd74^fl/fl^*: *n* = 7; *Cd74^fl/fl^*; *Lyz2‐Cre^ERT2^*: *n* = 7; *p* < .01). (B) Tumour growth curves for orthotopic OSCC models in *Cd74^fl/fl^; Lyz2‐Cre^ERT2^* and *Cd74^fl/fl^* mice (*Cd74^fl/fl^*: *n* = 7; *Cd74^fl/fl^*; *Lyz2‐Cre^ERT2^*: *n* = 7). (C, D) Comparison of recurrent tumour volumes and representative images of recurrence models in *Cd74^fl/fl^; Lyz2‐Cre^ERT2^* and *Cd74^fl/fl^* mice (*Cd74^fl/fl^*: *n* = 7; *^l^Cd74^fl/fl^*; *^ERT2^Lyz2‐Cre^ERT2^*: *n* = 6). (E) Representative haematoxylin and eosin (H&E) staining images in OSCC tongue injection models. (F, G) H&E and Pan‐CK IHC staining of draining lymph nodes from recurrence models. (H) Comparison of tumour areas in OSCC tongue injection models by H&E staining (*p* < .01). (I) Comparison of Pan‐CK positive areas quantification of draining lymph nodes from recurrence models (*p* < .01). (J, K) Multiplex immunofluorescence images for F4/80, CD74 and CD4 in OSCC tongue injection model and subcutaneous recurrent models.

To evaluate the impact of the CD74^hi^ TAMs dysfunction on tumour progression, we constructed OSCC tongue injection model and recurrence model of subcutaneously implanted tumour. CD74 deletion significantly accelerated tumour growth, especially in recurrent tumours (*p* < .01, Figure 4A–D). Haematoxylin and eosin (H&E) staining showed that the OSCC in the CD74 knockout group exhibited more invasion into the surrounding tissues, with ill‐defined boundaries (Figure 4E). Myeloid CD74 knock out increased tumour progress and LNM in recurrence model (Figure 4F–I), implying the crucial involvement of myeloid CD74 in anti‐tumour immunity and disease control. Moreover, CD74 knockout significantly reduced the contacts between CD74^hi^ TAMs and CD4^+^ T cells in the tongue injection oral cancer model, with significantly reduced CD4^+^ T‐cell infiltration (Figure 4J). Recurrent tumours exhibited further reduction (Figure 4K).

### 3.5 Mitochondrial metabolism modulated antigen presentation in TAMs

**FIGURE 5:**
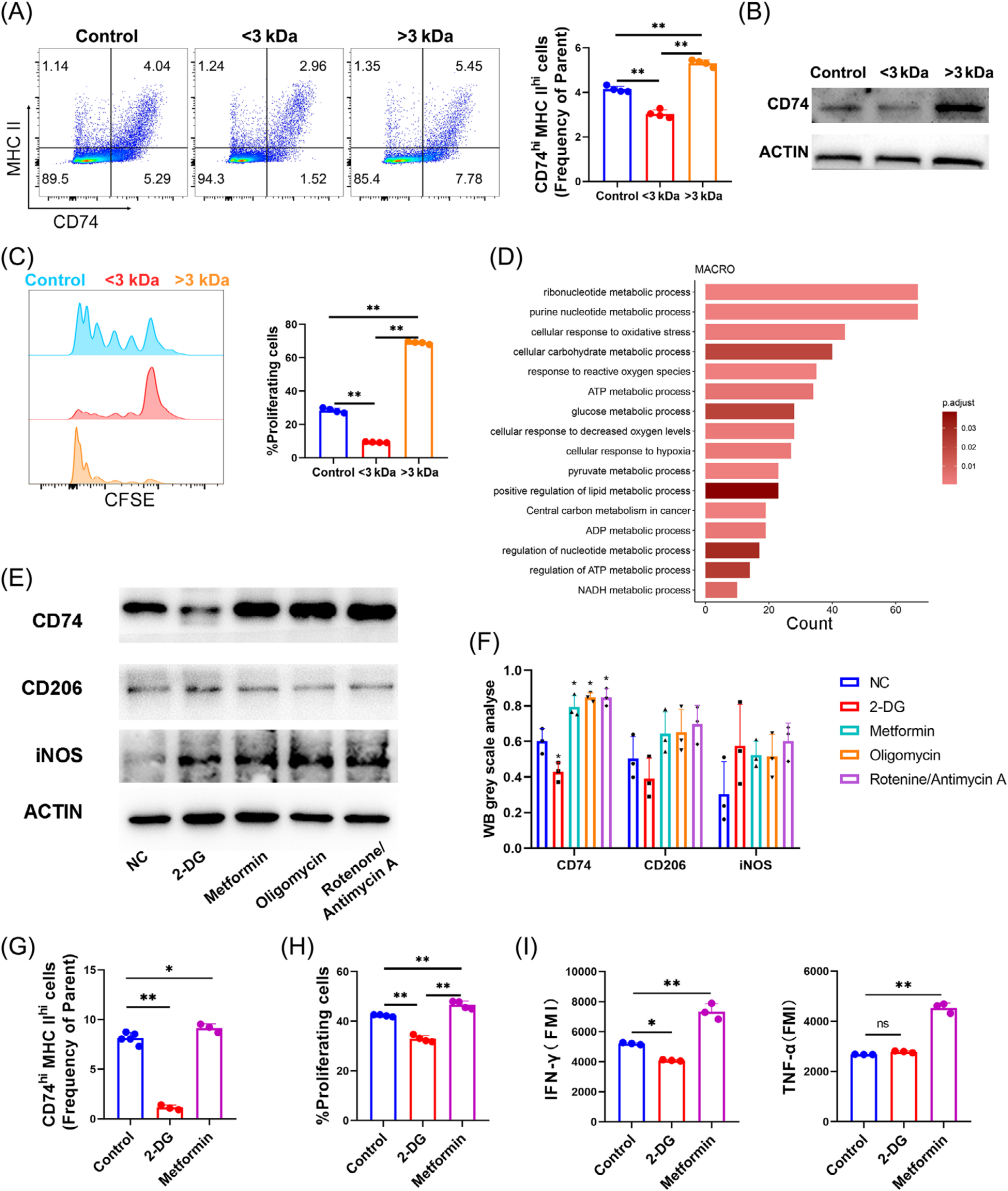
Metabolic reprogramming modulates antigen presentation in TAMs. (A) Metabolite fraction (<3 kDa) from the cell culture supernatant of MTCQ1 suppresses CD74^hi^ MHC II^hi^ macrophage differentiation, while the protein fraction (>3 kDa) promotes it. (B) The metabolite fraction reduces macrophage CD74 protein levels. (C) The metabolite fraction suppresses, while the protein fraction promotes, antigen presentation to OT‐II CD4^+^ T cells. (D) Upregulated genes in total TAMs from recurrent tumours enrich in pathways related to energy metabolism and oxidative stress. (E, F) Western blot shows that glycolysis inhibition with 2‐DG decreases CD74 expression, while ETC inhibition (metformin, oligomycin, rotenone/antimycin A) upregulates CD74; CD206 and iNOS are unchanged. (G) The proportion of CD74^hi^ MHC II^hi^ BMDMs influenced by 2‐DG or metformin. (H) The OT‐II CD4^+^ T‐cell proliferation via antigen presentation influenced by 2‐DG or metformin. (I) Metformin significantly stimulated BMDM‐induced IFN‐γ and TNF‐α secretion by OT‐II CD4^+^ T cells (*p < .05, **p < .01, ns: not significant).

To identify the potential mechanisms underlying CD74^hi^ TAMs downregulation in recurrent OSCC, we divided the MTCQ1 tumour cell‐conditioned medium into <3 kDa (metabolite fraction) and >3 kDa (protein fraction) components. Upon BMDM addition, the metabolite fraction significantly downregulated CD74 (*p* < .01, Figure 5A,B) and impaired the antigen‐presenting ability (Figure 5C). Conversely, CD74 expression was promoted in the protein fraction, indicating that soluble metabolites mediated CD74 suppression.

We then performed differential gene expression analysis using single‐cell transcriptomic data from patient tissues. Gene ontology (GO) analysis of upregulated genes in CD74^hi^ TAMs from recurrent tumours revealed OXPHOS, mitochondrial electron transport chain (METC), cytochrome c oxidase activity and ATP‐coupled proton transport pathways as the significantly enriched terms (Figure S6A,B). Pathway analysis across all TAMs further revealed increased oxidative stress responses, purine metabolism, ATP metabolism and pyruvate metabolism in recurrent tumours (Figure 5D), suggesting the involvement of mitochondrial metabolic reprogramming in the functional suppression of CD74 expression.

To establish whether TAMs metabolic activity directly affects CD74 expression, we treated TAMs isolated from murine OSCC tumours with glycolysis (2‐DG) and mitochondrial OXPHOS (metformin, oligomycin, rotenone/antimycin A) inhibitors. According to the WB analysis results, glycolytic inhibition with 2‐DG significantly downregulated CD74. Conversely, mitochondrial respiratory chain activity inhibition markedly upregulated CD74. Notably, the expression of canonical polarisation markers (CD206, iNOS) remained unchanged, indicating that the above effects on CD74 expression were independent of the polarisation state (Figure 5E,F).

Flow cytometry analysis of 2‐DG or metformin‐treated BMDMs further confirmed that mitochondrial inhibition upregulated CD74^hi^ MHC II^hi^ cells (*p* < .01 and *p* = .038, respectively, Figures 5G and S6C). Functionally, metformin treatment significantly enhanced the capacity of BMDMs to present antigens to OT‐II CD4^+^ T cells (*p* < .01, Figure 5H), promoting T‐cell proliferation and cytokine (IFN‐γ, TNF‐α) secretion (*p* < .01, Figures 5I and S6D). Conversely, 2‐DG impaired T‐cell activation and IFN‐γ production but had no significant effect on TNF‐α.

### 3.6 Extracellular lactate suppresses mitochondrial metabolism and impaired the antigen‐presenting function of TAMs

**FIGURE 6:**
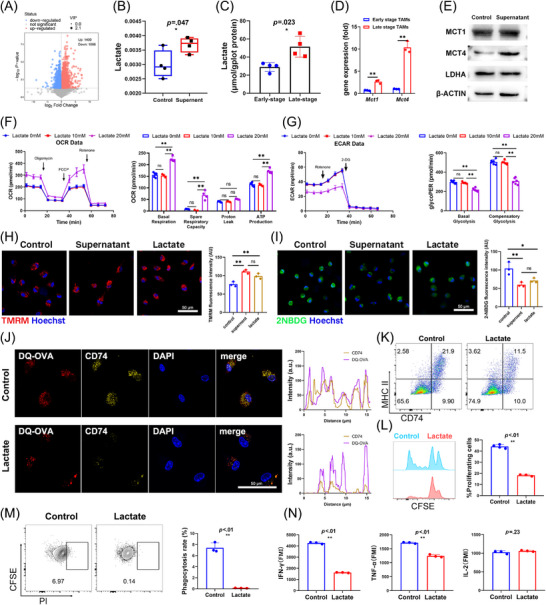
Extracellular lactate promotes oxidative phosphorylation, thus suppresses antigen presentation in TAMs. (A) The volcano plot shows the differential metabolites between TAMs (simulated by MTCQ1 cell supernatant) and BMDMs. (B) Lactate is the only differential metabolite related to mitochondrial metabolism. (C) Lactic acid accumulation is significantly increased in the late stage compared to the early stage of the mouse tumour‐bearing model (*p* = .023). (D) The RNA of TAMs shows a significant increase in the transcription of *Mct1* and *Mct4* in the late stage of the tumour‐bearing model (*p* < .05). (E) The protein expression of MCT1, MTC4 and LDHA in TAMs (simulated by MTCQ1 cell supernatant). (F) Lactate at 20 mM significantly enhanced mitochondrial respiration in BMDMs. (G) Lactate at 20 mM markedly inhibited glycolytic rate in BMDMs. (H, I) Representative fluorescence images of TMRM staining and 2NBDG uptake assays. Scale bar: 50 µm. *p < .05, **p < .01, ns: not significant. (J) Representative fluorescence images of DQ‐OVA and CD74 expression and co‐localisation analysis (right). Exogenous lactate stimulation significantly reduced DQ‐OVA peptide abundance, CD74 expression and their co‐localisation. Scale bar: 50 µm. (K) Lactate decreased the proportion of CD74^hi^ MHC II^hi^ macrophages (*p* < .01). (L) Proliferation of CD4^+^ T cells after co‐culture with antigen‐incubated TAMs. Exogenous lactate significantly suppressed exogenous antigen presentation by TAMs (*p* < .01). (M) Exogenous lactate markedly inhibited phagocytic activity of macrophages (*p* < .01). (N) Lactate attenuated IFNγ and TNFα secretion by CD4^+^ T cells following co‐culture with antigen‐presenting macrophages (*p* < .01).

Metabolomics profiling of tumour‐conditioned medium‐treated BMDMs revealed central carbon metabolism downregulation and upregulation of pantothenate and CoA biosynthesis pathways (Figure S6E). Among the differentially abundant metabolites, lactate emerged as a key candidate (*p* = .047), aligning with the known effects of tumour‐derived lactate in immunosuppression (Figure 6A,B).

Lactate concentrations were further assessed in early and late‐stage murine OSCC tumours, revealing significant lactate accumulation with tumour progression (*p* = .023, Figure 6C). Additionally, TAMs from late‐stage tumours showed the upregulation of lactate transporters *Mct1 and Mct4*. TAMs stimulated by MTCQ1 supernatant also upregulated MCT1, while lactate dehydrogenase A (LDHA) expression remained unchanged (Figure 6D,E), thus supporting a role for extracellular (rather than intracellular) lactate in macrophage function modulation.

To directly assess lactate effects, BMDMs were treated with exogenous lactate (10 mM, 20 mM). Seahorse assays were then performed, revealing that 20 mM lactate significantly increased basal respiration, spare respiratory capacity and adenosine triphosphate (ATP) production, while suppressing both basal and compensatory glycolytic rates (*p* < .01, Figure 6F,G). Low lactate (10 mM) concentrations exerted no significant influence. Furthermore, both lactate and tumour‐conditioned medium significantly increased the mitochondrial membrane potential (MMP detected by tetramethylrhodamine methyl ester perchlorate (TMRM) staining) and suppressed glucose uptake (detected by 2‐(N‐(7‐nitrobenz‐2‐oxa‐1,3‐diazol‐4‐yl)amino)‐2‐deoxyglucose (2‐NBDG) staining. in BMDMs (*p* < .01 and *p* < .05, respectively, Figure 6H,I), indicating a metabolic shift towards and away from OXPHOS and glycolysis, respectively.

Subsequently, we explored the influence of lactate on the antigen‐presenting function of TAMs. Notably, DQ‐OVA staining of lactate‐treated TAMs showed decreased antigen degradation and CD74 downregulation, indicating impaired antigen processing (Figure 6J). Functional assays further confirmed that lactate suppressed the phagocytic capacity (Figures 6M and S7A) and reduced the proportion of CD74^hi^ MHC II^hi^ macrophages (Figure 6K). Additionally, lactate markedly impaired BMDMs’ antigen presentation, reduced T‐cell proliferation and activation (Figure 6L). Flow cytometry revealed significant downregulation of IFN‐γ and TNF‐α in T cells (*p* < .01, Figures 6N and S7C). Enzyme‐linked immunosorbent assay (ELISA) analysis of culture supernatants showed reduced IFN‐γ, TNF‐α and IL‐2 secretion (Figure S7B). Collectively, these findings confirm that the tumour‐derived lactate could reprogram macrophage metabolism and suppress CD74‐dependent antigen presentation, ultimately impairing T‐cell‐mediated immune responses.

### 3.7 Lactate inhibited CD74 transcription via C/EBPα acetylation

**FIGURE 7:**
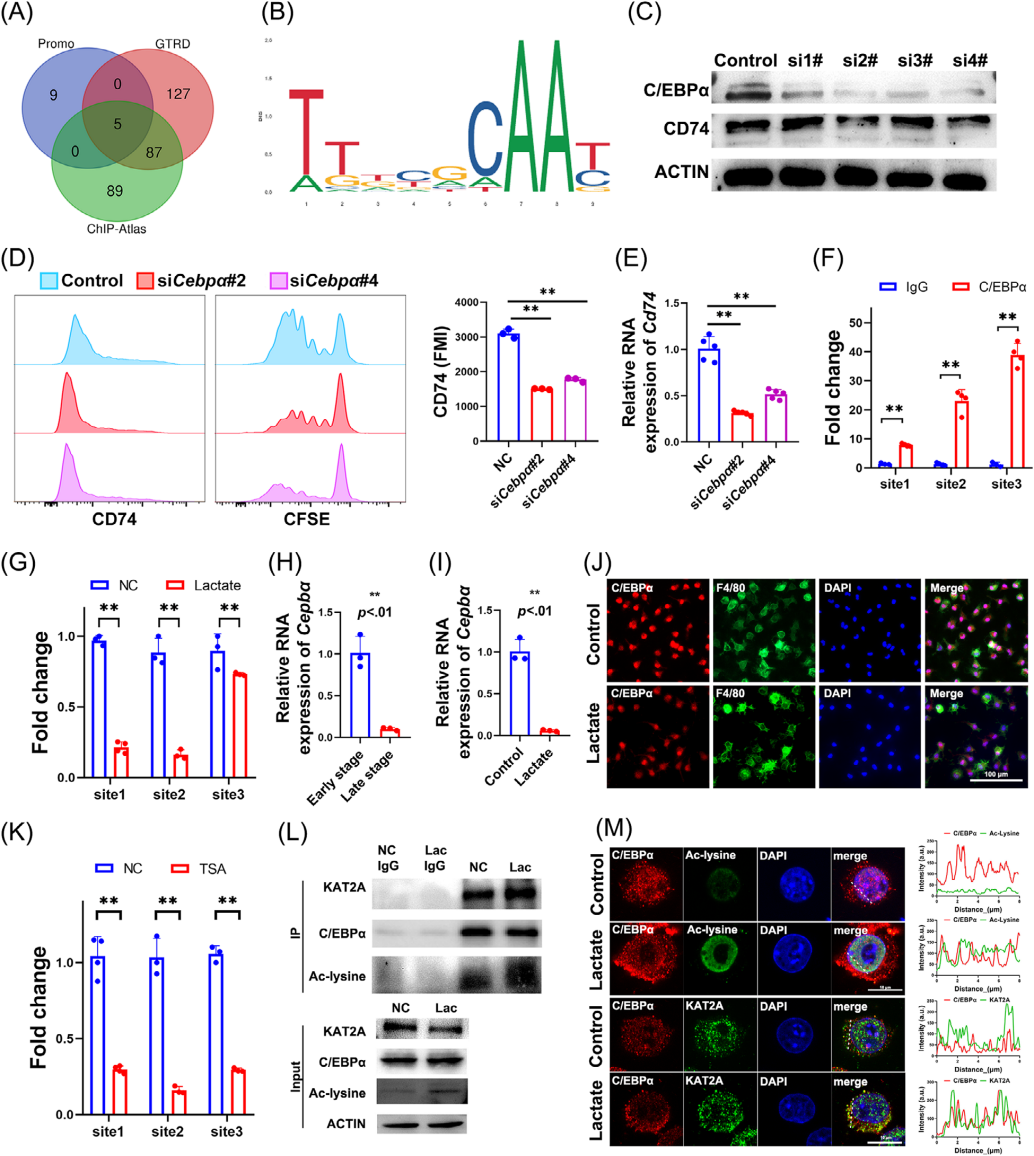
Lactate downregulates CD74 via C/EBPα acetylation. (A) Venn diagram of transcription factors predicted for *Cd74* by the PROMO, GTRD and ChIP‐Atlas databases, yielding five possible candidates: YY1, FOS, C/EBPβ, JUN and C/EBPα. (B) Sequence logo of the predicted DNA binding sites for C/EBPα on the *Cd74* promoter. (C) Four siRNAs targeting C/EBPα were constructed. Silencing C/EBPα expression in BMDMs with si2# and si4# significantly reduced CD74 protein levels. (D) Flow cytometry shows that C/EBPα silencing significantly decreases CD74 protein levels in BMDMs and reduces OT‐II CD4^+^ T‐cell activation and proliferation (*p* < .05). (E) C/EBPα silencing significantly lowers *Cd74* mRNA levels in BMDMs (*p* < .05). (F) CUT&RUN was performed to confirm the binding of C/EBPα to its consensuses (site1, site2 and site3) in the *Cd74* promoter in BMDMs (*p* < .01). (G) CUT&RUN assays revealed a significant reduction in C/EBPα enrichment at the *Cd74* promoter regions upon lactate stimulation (*p* < .01). (H) *C/ebpα* mRNA levels are significantly lower in TAMs from late‐stage tumours compared to early‐stage (*p* < .01). (I) Lactate significantly reduces *C/ebpα* mRNA levels in TAMs (*p* < .01). (J) Immunofluorescence images show that lactate downregulates C/EBPα in BMDMs. Scale bar: 100 µm. (K) CUT&RUN assays revealed the decreased enrichment of C/EBPα at the *Cd74* promoter after the treatment with TSA, the deacetylase inhibitor (*p* < .01). (L) Co‐IP confirmed the exogenous lactate stimulation enhanced the binding of C/EBPα to Ac‐lysine and KAT2A protein. (M) Representative cellular immunofluorescence images of C/EBPα, Ac‐lysine and KAT2A. Acetylated lysine expression was upregulated after lactate stimulation, with the increased co‐localisation of C/EBPα with Ac‐lysine and KAT2A. Scale bar: 10 µm.

To establish the upstream regulatory mechanism through which lactate suppresses CD74 expression in TAMs, BMDMs were treated with increasing lactate concentrations. According to the WB analysis results, low lactate concentrations upregulated CD74 in resting macrophages, while concentrations >10 mM significantly suppressed CD74 expression (Figure S8A). Based on prior metabolic findings, 20 mM lactate was selected as the standard for subsequent experiments. Quantitative real‐time polymerase chain reaction (RT‐qPCR) revealed that CD74 mRNA levels were significantly reduced upon lactate exposure (*p* < .01, Figure S8B), while cycloheximide (CHX) chase assays showed that CD74 protein stability was not decreased—but rather modestly increased (Figure S8C)—suggesting that CD74 suppression occurs primarily at the transcriptional level rather than through post‐translational degradation. To determine the transcriptional regulators of CD74 expression, we intersected predictions from PROMO: ALGGEN promoter analysis and TF binding site prediction (PROMO), Gene Transcription Regulation Database (GTRD), and ChIP‐Atlas database, yielding five possible candidates: YY1, FOS, C/EBPβ, JUN and C/EBPα (Figure 7A). Among these, lactate treatment only downregulated C/EBPα protein expression in a manner consistent with CD74 repression (Figure S9). Binding site prediction using the JASPAR database revealed C/EBPα motifs in the CD74 promoter region (Figure 7B).

The CCAAT/enhancer‐binding protein alpha (C/EBPα) is known to regulate myeloid lineage development and M‐CSF/GM‐CSF signalling. We evaluated C/EBPα and CD68 expression in OSCC patient tumour sections and found that patients with high C/EBPα expression exhibited a more favourable prognosis (Figure S9B,C). Correlation analysis showed that although C/EBPα expression was not significantly associated with CD8^+^ cells infiltration, its expression in TAMs was positively correlated with CD74 levels (*r* = .403, *p* < .01; Figure S9D). To confirm the connection between CD74 and C/EBPα, we performed C/EBPα knockdown in BMDMs. C/EBPα knockdown significantly reduced CD74 protein (*p* < .01, Figure 7C,D) and mRNA expression (*p* < .01, Figure 7E), and weakened the ability of BMDMs to activate OT‐II CD4^+^ T cells (Figure 7D). CUT&RUN was performed to confirm the binding of C/EBPα to its consensuses (site1, site2 and site3) in the *Cd74* promoter in BMDMs (*p* < .01, Figure 7F).

We then evaluate the influence by lactate in tumour environment. We found that lactate treatment significantly impaired C/EBPα binding to the *Cd74* promoter (Figure 7G), suggesting that lactate disrupts *Cd74* transcription via C/EBPα activity inhibition. Additionally, expression of *C/ebpα* was also reduced in TAMs from late‐stage tumours and after lactate stimulation (Figure 7H–J).

Lactate metabolism via mitochondrial OXPHOS promotes acetyl‐CoA generation, a key substrate for protein acetylation. Additionally, lysine acetyltransferase KAT2A could acetylate C/EBPα by disrupting its DNA binding and transcriptional activity. We found that lactate exposure significantly increased global protein acetylation in BMDMs in a dose‐dependent manner (Figure S10A). Co‐immunoprecipitation (Co‐IP) further confirmed that C/EBPα interacts with acetylated lysine (Ac‐lysine) residues and KAT2A (Figure S10B). Additionally, in CUT&RUN assays, treatment with the histone deacetylase (HDAC) inhibitor TSA enhanced C/EBPα acetylation and markedly decreased its binding to the *Cd74* promoter (Figure 7K), indicating that C/EBPα acetylation could directly impair *Cd74* transcription. Moreover, Co‐IP and IF analysis of lactate‐treated BMDMs showed increased binding and co‐localisation of C/EBPα with KAT2A and Ac‐lysine residues (Figure 7L,M), confirming that exogenous lactate promotes C/EBPα acetylation and transcriptional inactivation.

To further assess the in vivo relevance of C/EBPα lactylation in OSCC, we examined the expression of C/EBPα, CD74 and Ac‐lysine in macrophages from the primary and recurrent tumour model (Figure S11A). Recurrent tumours exhibited increased infiltration of CD74^lo^ TAMs, which showed enhanced co‐localisation of C/EBPα with Ac‐lysine.

Treatment with the HDAC activator iTSA increased CD74 expression and restored antigen presentation function in tumour cell supernatant‐stimulated BMDMs in vitro (Figure S11B,C). Consistently, in vivo iTSA administration during tumour establishment significantly increased the infiltration of CD74^hi^ TAMs and reduced tumour growth (Figure S11D,E). Collectively, these data suggest that targeting C/EBPα lactylation may represent a potential therapeutic strategy in OSCC.

## 4 DISCUSSION

As the most abundant immune cell population within the TME, TAMs are often located in close spatial proximity to tumour cells, enabling them to continuously capture tumour‐derived neoantigens and mediate local antigen presentation and immune activation within the tumour niche.^29^, ^30^, ^31^ Although dendritic cells (DCs) are widely recognised as the classical professional APCs, they typically need to migrate to the draining lymph nodes to prime T cells, whereas TAMs predominantly reside within the tumour and can mediate antigen presentation locally. The antigen‐presenting capacity and immunoregulatory roles of TAMs have recently gained notable scholarly attention. In a murine model of glioma, TAMs—rather than DCs or tumour cells—were identified as the predominant source of T‐cell antigen priming.^10^ Furthermore, impaired antigen presentation by TAMs results in chronic, low‐level cross‐presentation to CD8^+^ T cells, which ultimately promotes T‐cell exhaustion. Notably, disrupting this dysfunctional process enhances the therapeutic efficacy of anti‐PD‐1 treatment across multiple tumour models.^10^ scRNA‐seq revealed that in addition to HLA‐DRA and other MHC II components, CD74^hi^ TAMs also express co‐stimulatory molecules such as CD80 and CD86, implying their ability to deliver both signals 1 and 2 required for full T‐cell activation,^32^ thus highlighting TAMs as key regulators—rather passive participants—of adaptive immune responses.

As a critical chaperone of MHC class II molecules, CD74 governs antigen presentation through a tightly regulated multi‐step process.^33^ For instance, CD74 trimers interact with newly synthesised MHC II α/β chains in the endoplasmic reticulum (ER), preventing premature endogenous peptide loading via peptide‐binding groove occlusion and directing complex trafficking to the endosomal pathway through sorting signals.^34^ Additionally, CD74 is progressively degraded within the endosomes, and exogenous peptides often replace its residual CLIP peptide, creating stable MHC II–peptide complexes ready for T‐cell recognition.^35^, ^36^ In our research, the infiltration of CD74^hi^ TAMs correlated with a favourable prognosis. Besides impairing T‐cell activation, targeted deletion of CD74 in macrophages also disrupted phagocytosis and antigen processing. Although this mechanism remains poorly characterised, it could be attributed to CD74 isoforms—including p33, p35, p41 and p43. For example, the p41 isoform contains a thyroglobulin domain that interacts with cathepsin L^37^; hence, we hypothesised that CD74 loss may modulate lysosomal protease activity, thus reducing peptide hydrolysis efficiency and impairing antigen processing. However, given the limited sample size in the current study, further validation in larger and independent cohorts will be required.

Despite its central role, the immunological function of CD74 in tumours remains poorly defined.^36^ Multiple single‐cell studies reported high CD74 expression in immune subsets. For instance, Basha et al. found a subset of HLA‐DR^+^CD74^+^ neutrophils that may promote a ‘hot’ TME via enhanced antigen presentation.^38^ However, in some malignancies, heavy CD74^+^ immune cell infiltration was paradoxically associated with poor prognosis.^39^ CD74 has also been reported to contribute to tumour growth and immune evasion through interaction with macrophage migration inhibitory factor (MIF).^40^ Herein, the infiltration of CD74^hi^ TAMs within the OSCC microenvironment correlated with a favourable prognosis. However, with OSCC progression and recurrence, the proportion of CD74^hi^ TAMs declined significantly. Moreover, CD74 loss severely impaired the antigen‐presenting capacity of macrophages, suppressed T‐cell immune activation and accelerated tumour progression, thereby establishing a vicious cycle. In addition to MHC II–restricted CD4^+^ T‐cell priming, macrophages can also support CD8^+^ T‐cell responses through antigen cross‐presentation. Although cross‐presentation is primarily mediated via MHC class I, it shares upstream processes with MHC II presentation (e.g., phagocytosis and antigen processing); therefore, CD74 loss‐induced defects in antigen uptake and processing may also indirectly compromise CD8^+^ T‐cell activation.^33^ Nevertheless, whether and how CD74‐dependent pathways in TAMs influence CD8^+^ T‐cell cross‐priming in OSCC will require further investigation.

The TAMs metabolism–immune function relationship has recently emerged as a central focus of TME research. Classically activated M1 macrophages exhibited suppressed mitochondrial OXPHOS, relying instead on glycolysis for ATP generation, while producing ROS that amplify inflammation. Conversely, alternatively activated M2 macrophages triggered mitochondrial biogenesis through the AMPK/PGC‐1α pathway, enhanced fatty acid oxidation and OXPHOS, and maintained lasting mitochondrial networks to support tissue repair and immune suppression.^41^ Moreover, metabolic reprogramming could reciprocally shape macrophage function. Specifically, mitochondrial transplantation can reprogram TAMs and restore immunostimulatory capacity, enhancing T‐cell activation and tumour suppression.^42^ Herein, mitochondrial dysregulation in CD74^hi^ TAMs from recurrent OSCC tumours correlated with impaired antigen presentation. Furthermore, METC inhibition with metformin and oligomycin upregulated CD74, whereas inhibition with 2‐DG suppressed CD74 expression levels. While previous studies linked mitochondrial dysfunction to AMPK activation and macrophage polarisation,^43^ our findings revealed that neither OXPHOS inhibition nor glycolytic blockade significantly altered MHC II expression, implying that CD74 regulation operates independently of canonical M1/M2 polarisation pathways.

Multiple tumour‐derived factors, including metabolic byproducts, hypoxia, immunosuppressive cytokines and surface ligands, have been established to converge to inhibit TAMs activation.^44^ Lactate, once considered a metabolic waste, is presently recognised as a key signalling molecule that could directly activate METC and initiate histone and protein lactylation, thus promoting metabolic reprogramming.^45^ Lactate could infiltrate mitochondria through monocarboxylate transporters (MCTs), wherein it would be converted into pyruvate via LDH, fuelling the TCA cycle and OXPHOS.^46^ Notably, lactate could enhance mitochondrial respiration and ATP production independently of pyruvate conversion, thus reducing reliance on glucose metabolism^47^—a phenomenon our findings validated. Specifically, the tumour‐conditioned medium upregulated mitochondrial activity and MCT expression in TAMs, without significantly altering LDHA expression.

Lactate accumulation in the TME also significantly suppressed antigen processing—a phenomenon attributable to excessive lactate uptake‐induced metabolic competition and mitochondrial overload, leading to ETC electron leakage and ROS accumulation.^48^ High ROS levels could induce ER stress (ERS) and inhibit APM components, such as transporter associated with antigen processing (TAP), ultimately reducing antigen peptide generation.^49^ Moreover, lactate‐induced extracellular acidification could impair lysosomal protease activity, attenuating peptide degradation and exacerbating immunosuppressive effects.

Besides metabolic interference, lactate may also modulate antigen presentation via epigenetic mechanisms.^50^ Brand et al. reported that lactate‐derived acetyl‐CoA may alter histone acetylation in macrophages, suppressing the enhancer activity of pro‐inflammatory genes during M1 polarisation.^28^ Herein, lactate downregulated both CD74 mRNA and protein levels. Moreover, CHX chase assays revealed that lactate increased CD74 protein stability—a phenomenon attributable to lactylation‐mediated suppression of ubiquitination and proteasomal degradation.^51^ Despite stabilising CD74 transiently, this process may prevent CD74 dissociation from the MHC II groove, obstructing proper antigen loading and delivery.^52^

According to reports, C/EBPα is subject to post‐translational regulation by acetylation and phosphorylation.^53^, ^54^, ^55^, ^56^ Acetylation at lysine residues *K298* and *K302* by KAT2A neutralised the positive charges in the basic region of C/EBPα, reducing its DNA‐binding affinity, and limiting recruitment to gene promoters, including that of the G‐CSF receptor, ultimately repressing transcriptional activation and granulocyte differentiation.^56^ Our findings supported this model. Specifically, while lactate stimulation enhanced C/EBPα acetylation and its interaction with KAT2A, HDAC inhibition significantly reduced C/EBPα binding to the *Cd74* promoter. Although HDAC inhibitors are under active clinical development,^57^ selective agonists or inhibitors targeting the acetylation of TFs remain underexplored. Therefore, future studies should focus on developing C/EBPα acetylation modulators to reverse TAMs dysfunction and restore effective antigen presentation in the TME.

## AUTHOR CONTRIBUTIONS

**Mengyao Wang**: Conceptualisation, Methodology, Software, Data curation, Writing – Original draft preparation, Writing – Review & Editing. **Mengqi Wang**: Methodology, Software, Visualisation, Investigation. **Zizhen Gong**: Methodology, Software, Visualisation, Investigation, Writing – Review & Editing. **Fanrui Zeng**: Methodology, Software, Investigation. **Zihui Ni**: Methodology, Software, Visualisation, Writing – Original draft preparation. **Rundong Zhai**: Methodology, Software, Visualisation. **Weiwen Zhu**: Conceptualisation, Supervision, Writing – Review & Editing. **Jiayi Zhang**: Conceptualisation, Methodology, Supervision. **Laikui Liu**: Conceptualisation, Methodology, Supervision.

## CONFLICT OF INTEREST STATEMENT

The authors declare no conflicts of interest.

Public scRNA‐seq datasets were retrieved from GEO under accession numbers GSE41613 and GSE65858.

All remaining data are available from the corresponding author upon reasonable request.

## ETHICS STATEMENT

The study protocol for human sample collection was reviewed and approved by the Ethics Committee of Nanjing Medical University (Approval No. 1029–848) and complied with the Declaration of Helsinki. Written informed consent was obtained from all participants. All animal experiments were approved by the Animal Ethics and Welfare Committee of Nanjing Medical University (IACUC‐230624).

## Supporting information



Supporting Information

## Data Availability

The scRNA‐seq datasets analysed in this study include previously published data from our group, which have been deposited in the GSA for Human database at the National Genomics Data Center (NGDC) under accession number HRA010576. In addition, the newly generated scRNA‐seq data have been deposited in the same repository under accession number HRA016848. Both datasets are publicly available through the NGDC GSA for Human portal (https://ngdc.cncb.ac.cn/gsa‐human/).
