# Supplementary material for: Lactate‐induced metabolic reprogramming of TAMs impairs antigen presentation capacity via C/EBPα–CD74 axis in oral squamous cell carcinoma
Source: Clin Transl Med. 2026 Mar 10;16(3):e70639. doi: 10.1002/ctm2.70639 (PMC12973165; doi:10.1002/ctm2.70639)
Supplement: Supplementary file 1 — Supporting Information [file CTM2-16-e70639-s001.docx]

**Supplementary Information**

**Experimental Section/Methods**

**Public scRNA-seq Data Extraction**

The expression matrices were extracted using the exprs() function, and clinical metadata were obtained via the pData() function within the GEOquery R package.

**Data Preprocessing and Quality Control (QC)**

To ensure inter-platform consistency, the following quality control measures were implemented:

Normalization: Quantile normalization was applied to eliminate systematic biases between arrays.

Probe Mapping: Probe IDs were mapped to official Gene Symbols using the hgu133plus2.db and illuminaHumanv4.db annotation packages, respectively.

Consistency Validation: The distribution of expression values across all samples was visualized using the boxplot function in R to ensure aligned medians. In cases where multiple probes mapped to a single gene, the mean expression value was utilized.

**Feature Gene Expression Analysis of Single-cell sequencing**

To identify marker genes for each sub-cluster, the FindAllMarkers function was employed. The resulting marker genes were then visualized using the DotPlot function to demonstrate their expression patterns across different clusters.

**Cell Proportion Analysis**

The proportion of each cell type within individual samples was calculated using the reshape2 package (v1.4.4). These proportions were subsequently visualized as bar plots using the ggplot2 package to illustrate the cellular composition across different groups.

**Monocle 2 Pseudotime Trajectory Analysis**

Pseudotime trajectory analysis was performed using the Monocle 2 package（v2.26.0）. Genes with a mean expression≥0.1 and an empirical dispersion greater than the fitted dispersion were selected as ordering genes to define the developmental trajectory. Dimensionality reduction was executed using the DDRTree algorithm, followed by pseudotime ordering of the cells. The cell differentiation trajectories were subsequently visualized using the plot_cell_trajectory function.

**Gene Ontology (GO) Enrichment Analysis**

Gene Ontology (GO) enrichment analysis was performed on the differentially expressed genes (DEGs) between the recurrent (R) and primary (T) groups within the MACRO cell clusters using the enrichGO function from the clusterProfiler package (v4.10.0). The enrichment results were subsequently visualized as bubble plots using the ggplot2 package (v3.4.4) to illustrate the significantly enriched biological processes, molecular functions, and cellular components.

**Survival analysis**

The prognostic impact of CD74^hi^T AMs on patients with oral squamous cell carcinoma (OSCC) was evaluated using the deconvolution analysis results of the CIBERSORTx algorithm applied to the public datasets GSE41613 and GSE65858, in combination with the Kaplan-Meier method and the Cox proportional hazards model.

**Cell culture**

The 4NQO-induced mouse tongue cancer cell line MTCQ1 (RRID: CVCL_A9X0) was purchased from the Bioresource Collection and Research Center on 7 June 2023. BMDMs were extracted from mouse bone marrow. OT-II CD4⁺ T cells used for antigen presentation experiments were isolated from the spleens of OT II mice and validated by flow cytometry. and validated by flow cytometry. Cells were maintained at 37 °C in a 5% CO2-humidified incubator and confirmed free of mycoplasma contamination.

**Isolation of mice TAMs**

A lysis buffer was prepared using deoxyribonuclease I (DNase I), hyaluronidase (HAase), and collagenase type IV (Cls IV). Tumor was dissected under sterile conditions and digested by lysis buffer. TAMs were isolated using F4/80 Magnetic cell separation and validated by flow cytometry.

**Phagocytosis Assays**

Macrophages were co-cultured with UV-induced apoptotic MTCQ1 tumor cells, which were labeled with PI and CFSE, respectively. Phagocytosis efficiency was quantified using flow cytometry and visualized under a fluorescence microscope.

**Antigen Processing Assays**

To assess antigen processing, CFSE-labeled macrophages were first pre-starved and subsequently incubated with DQ-OVA (10 μg/mL) for 2 hours. The resulting fluorescence intensity was examined through flow cytometry and confocal microscopy.

**Separation of metabolic components and protein components**

Transfer the cell culture medium to a 3 kDa ultrafiltration centrifuge tube, then collect the filtrate (components < 3 kDa) and retentate (components > 3 kDa). The filtrate is used for subsequent cell culture, while the retentate is diluted to the original concentration before being used for subsequent culture.

**Mitochondrial membrane potential estimation**

The cells are incubated with serum-free medium (containing TMRM) for 20-30 minutes. After washing, the cells are stained with Hoechst staining solution and observed.

**Glucose uptake assay**

The glucose uptake was evaluated by 2-NBDG. After a 6-hour starvation treatment with serum-free medium, the cells were cultured with a working solution containing 10% FBS and 100 μM 2NBDG for 30 minutes. After washing, the cells are stained with Hoechst staining solution and observed.

**Immunofluorescence Staining**

The cell smears were fixed with 4% PFA, permeabilized with 0.1% Triton X-100, and blocked with 5% BSA. This was followed by incubation with primary antibody, washing and further incubation with the fluorophore-conjugated secondary antibody. It was then counterstained DAPI for nuclei staining. It was mounted with the antifade medium and images were taken using a fluorescence microscope.

**Flow Cytometry**

Cells were extracted and prepared to a single-cell suspension. The Fc receptors were blocked and then cells were incubated with fluorescently labeled antibodies targeting the surface markers. For intracellular staining, the cells were fixed/permeabilized, then washed thoroughly. It was resuspended in a buffer and the stained cells were immediately analyzed using a flow cytometer.

**Western Blot**

Protein samples were resolved through SDS-PAGE gels and transferred to PVDF membranes via wet transfer at 200 mA for 40 minutes on ice. The membranes were blocked in 5% skim milk in TBST for 2 hours, incubated overnight at 4°C with primary antibodies, and then with the HRP-conjugated secondary antibodies for 1 hour at room temperature. Finally, the signals were detected using enhanced chemiluminescence (ECL).

**Figures and tables
Fig. S1.**

**
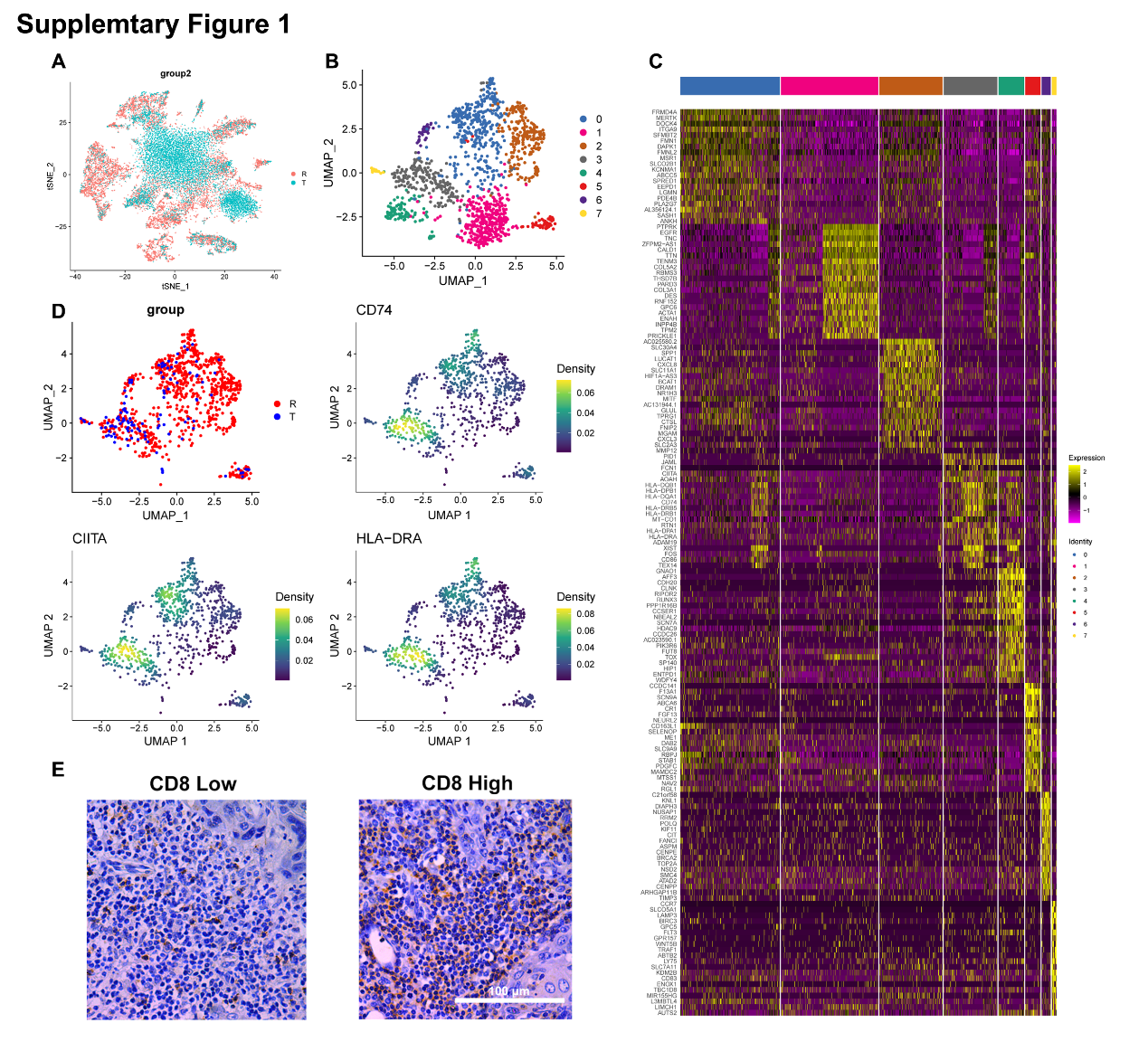
**

**(A)** UMAP plot shows the patients group of all clusters. **(B)** UMAP plot shows subclusters of myeloid cells. **(C)** Top 20 marker genes of subclusters in myeloid cells. **(D)** Expression Profile of Antigen Presentation Genes (CD74, CIITA, HLA-DRA) in Macrophage Subsets. **(E)** Representative IHC images of tumors with high and low CD8⁺ T cell infiltration. Scale bar: 100 μm.

**Fig. S2.**

**
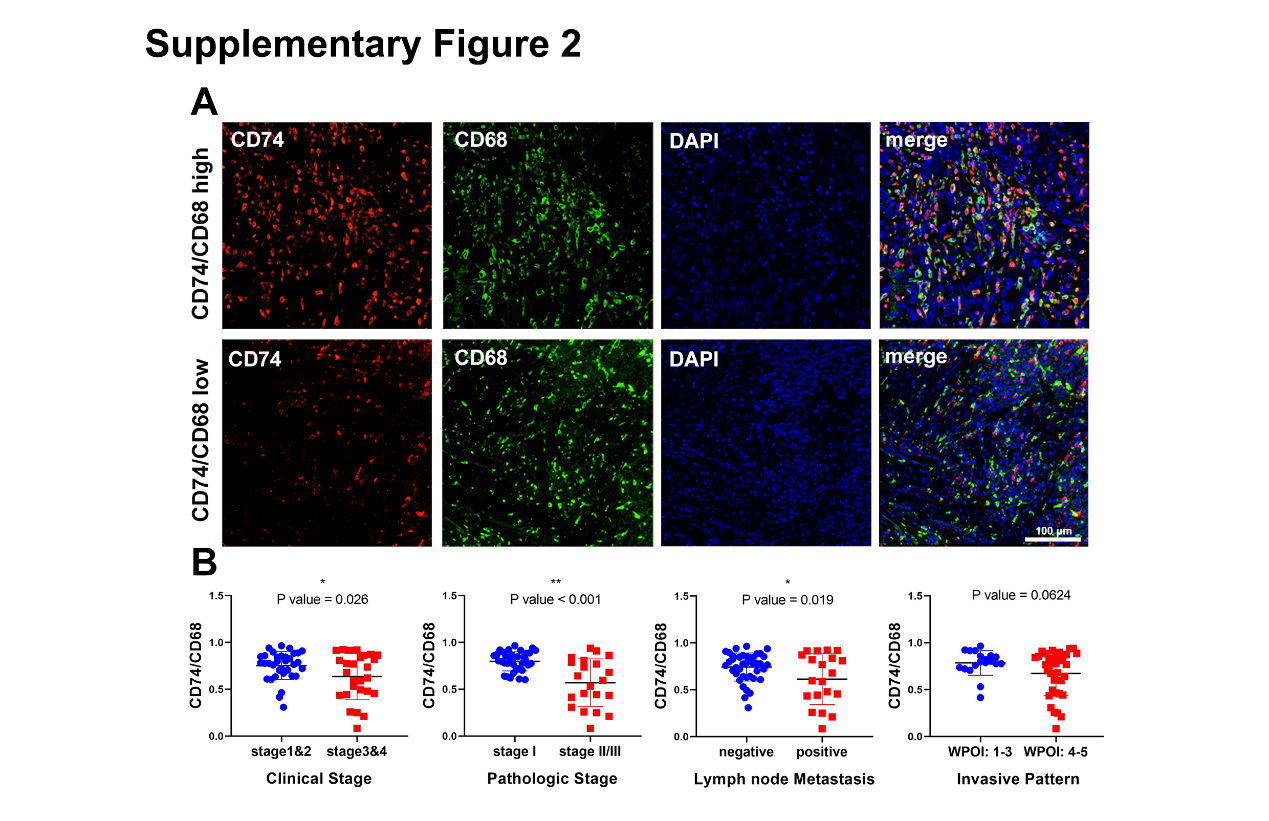
**

**(A)** Representative images of TAMs with high (top) and low (bottom) CD74 expression in OSCC tissues. Scale bar: 100 μm. **(B)** CD74/CD68 ratio scores show significant differences when grouped by lymph node metastasis, histological grade, and clinical stage (P = 0.019, < 0. 001, and 0.016, respectively).

**Fig.S3.**
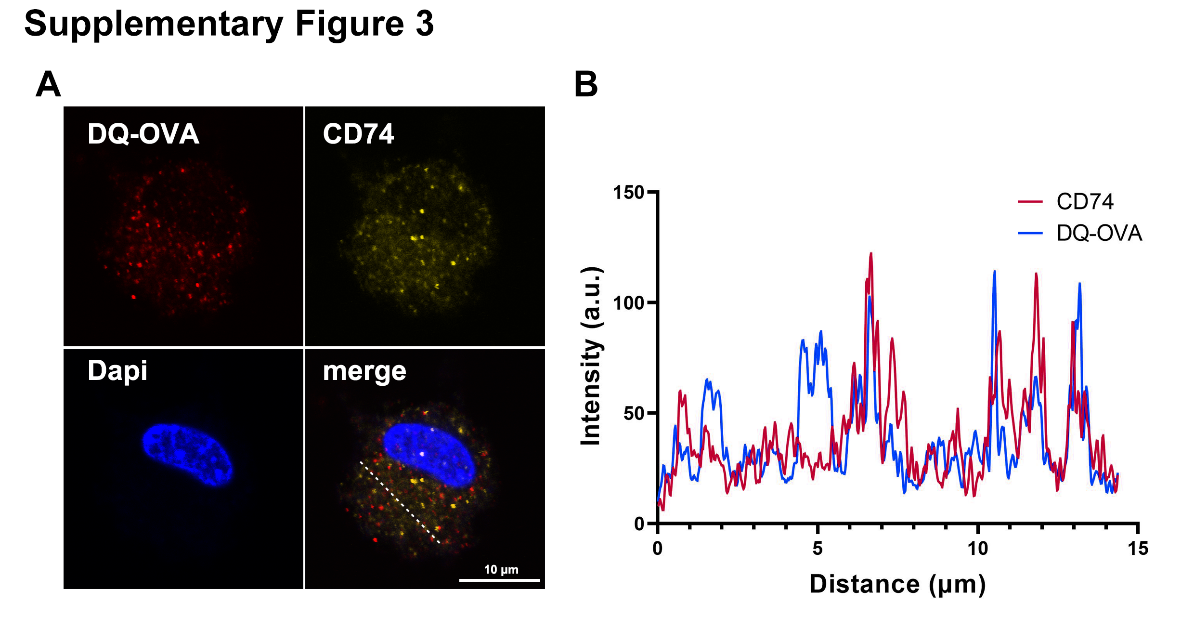


**(A–B)** Representative immunofluorescence images **(A)** and signal quantification **(B)** showing extensive colocalization of CD74 with processed antigen peptides inside TAMs, detected by DQ-OVA and CD74 staining. Scale bar: 10 μm.

**Fig. S4.**

**
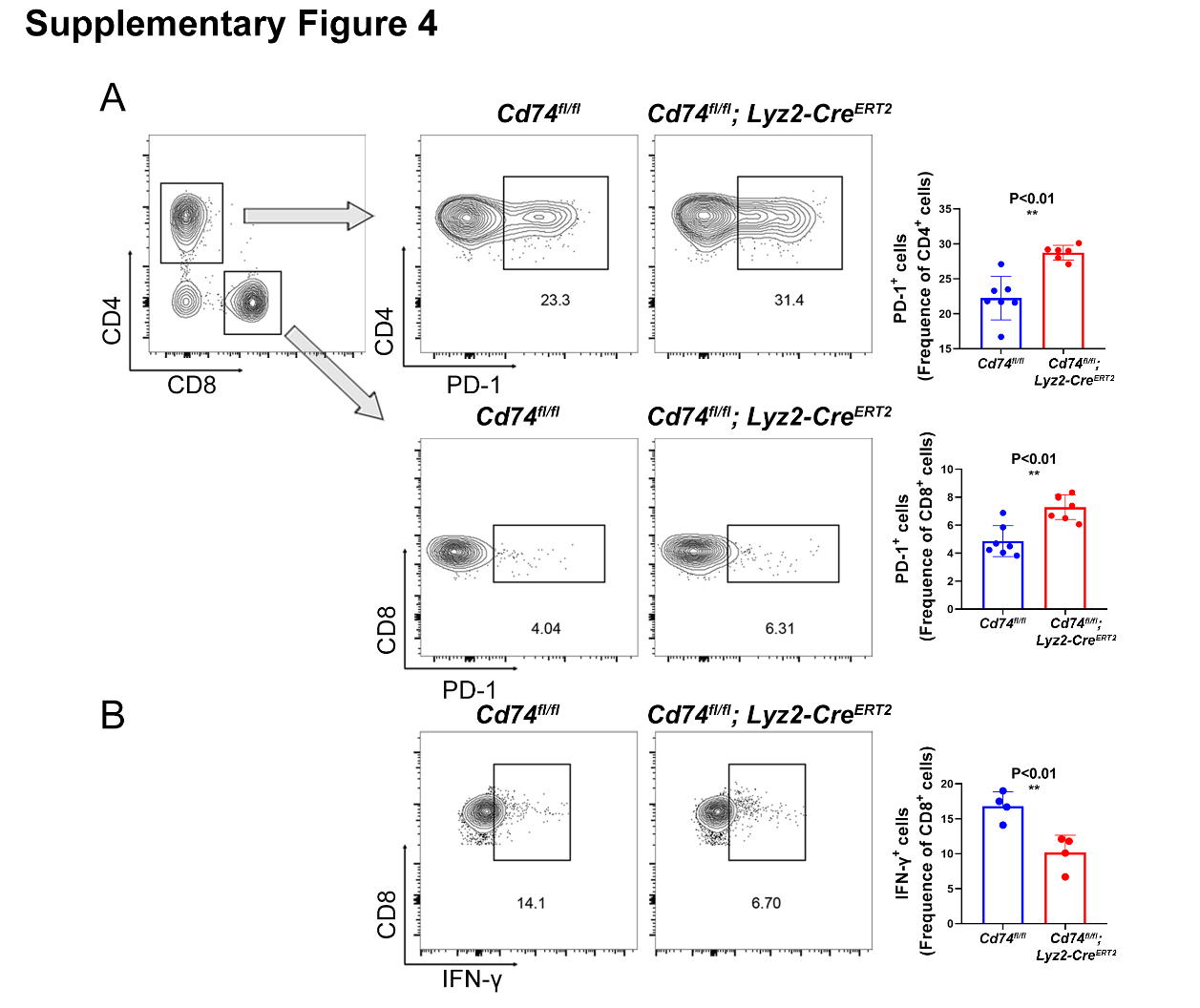
**

**(A)** Myeloid CD74 conditional knockout significantly increases PD-1 expression on splenic CD4⁺ and CD8⁺ T cells (both P < 0. 01). **(B)** CD74 deletion in myeloid cells significantly inhibits IFN-γ secretion by CD8⁺ T cells (P < 0. 01) (*CD74^fl/fl^*: n = 7; *CD74^fl/fl^; Lyz2-Cre^ERT2^*: n = 6).

**Fig. S5.**


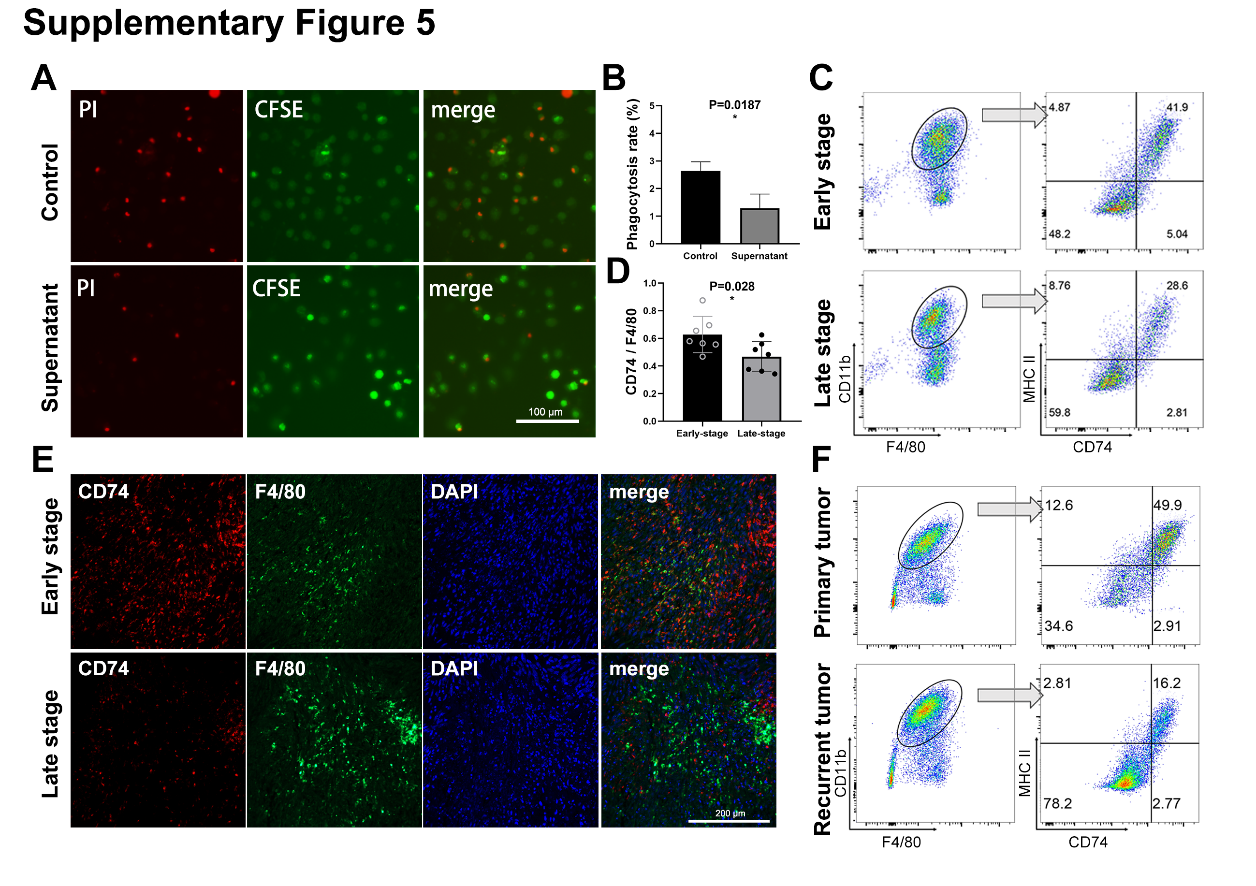


**(A–B)** Representative fluorescence microscopy images of phagocytosis assays. BMDMs show significantly reduced phagocytosis of apoptotic tumor cells after stimulation with MTCQ1-conditioned medium (P = 0. 0187). Scale bar: 100 μm. **(C)** Representative images of CD74^hi^ MHC II^hi^ TAMs infiltration in early stage and late stage OSCC subcutaneous tumor models. **(D–E)** CD74^hi^ TAMs infiltration is significantly decreased in late stage tumors compared to early stage (P = 0. 028; Early stage: n = 7; Late stage: n = 7). Scale bar: 200 μm. **(F)** Representative images comparing CD74^hi^ MHC II^hi^ cell proportions in TAMs from primary versus recurrent tumors.

**Fig. S6.**

**
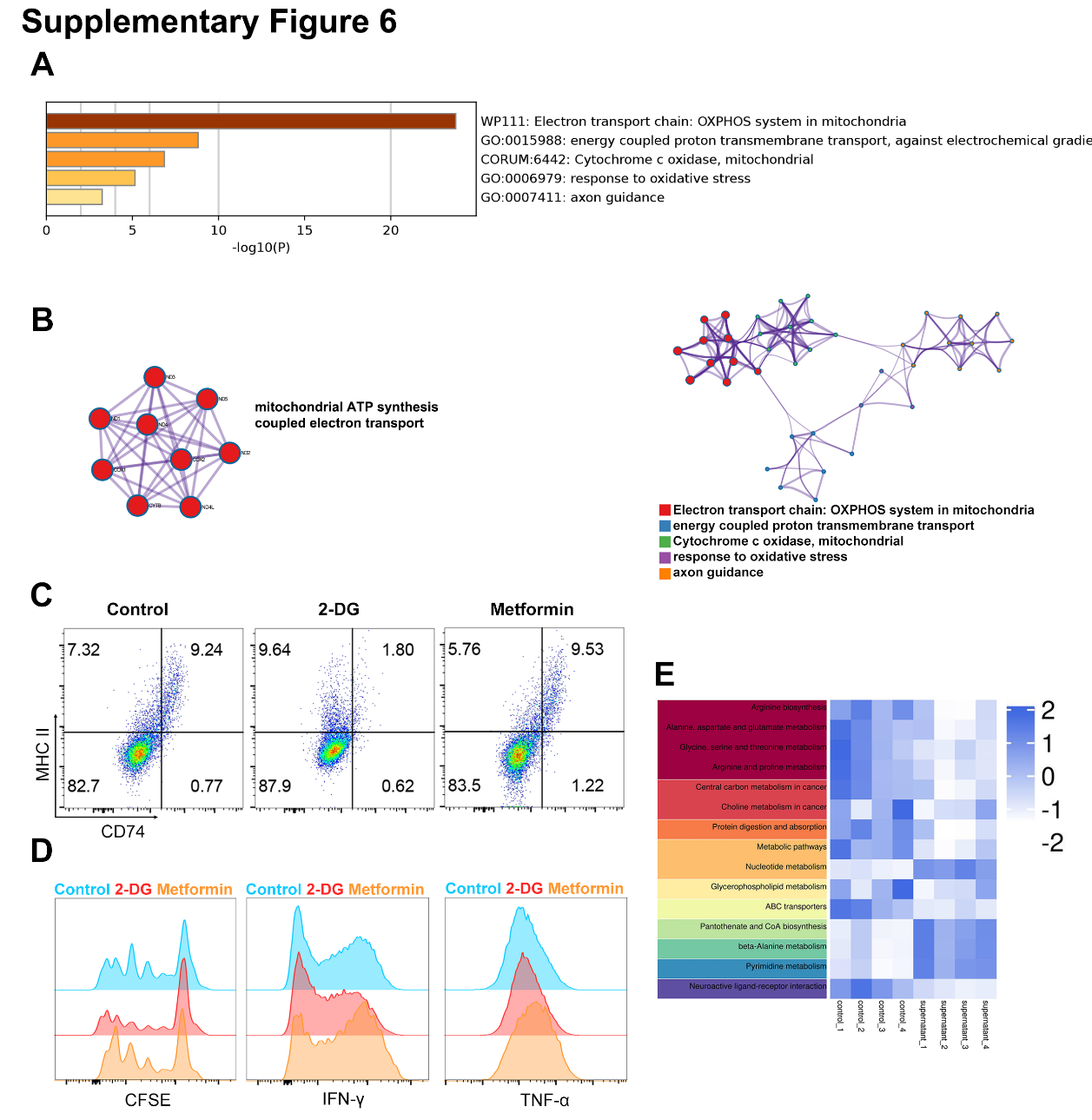
**

**(A)** Metascape analysis shows the enriched pathways of upregulated DEGs in CD74^hi^ TAMs from recurrent OSCC. **(B)** Network of enriched pathways and MCODE analysis for upregulated genes. **(C)** Representative images showing changes in the proportion of CD74^hi^ MHC II^hi^ cells in BMDMs treated with 2-DG or metformin. **(D)** Representative images showing proliferation of OT-II CD4⁺ T cells activated by antigen presentation after 2-DG or metformin treatment of BMDM and effects on T cell’ IFN-γ and TNF-α secretion. **(E)** Metabolomics results show downregulation of central carbon metabolism and upregulation of pantothenate and CoA biosynthesis in macrophages stimulated with tumor-conditioned medium.

**Fig. S7.**


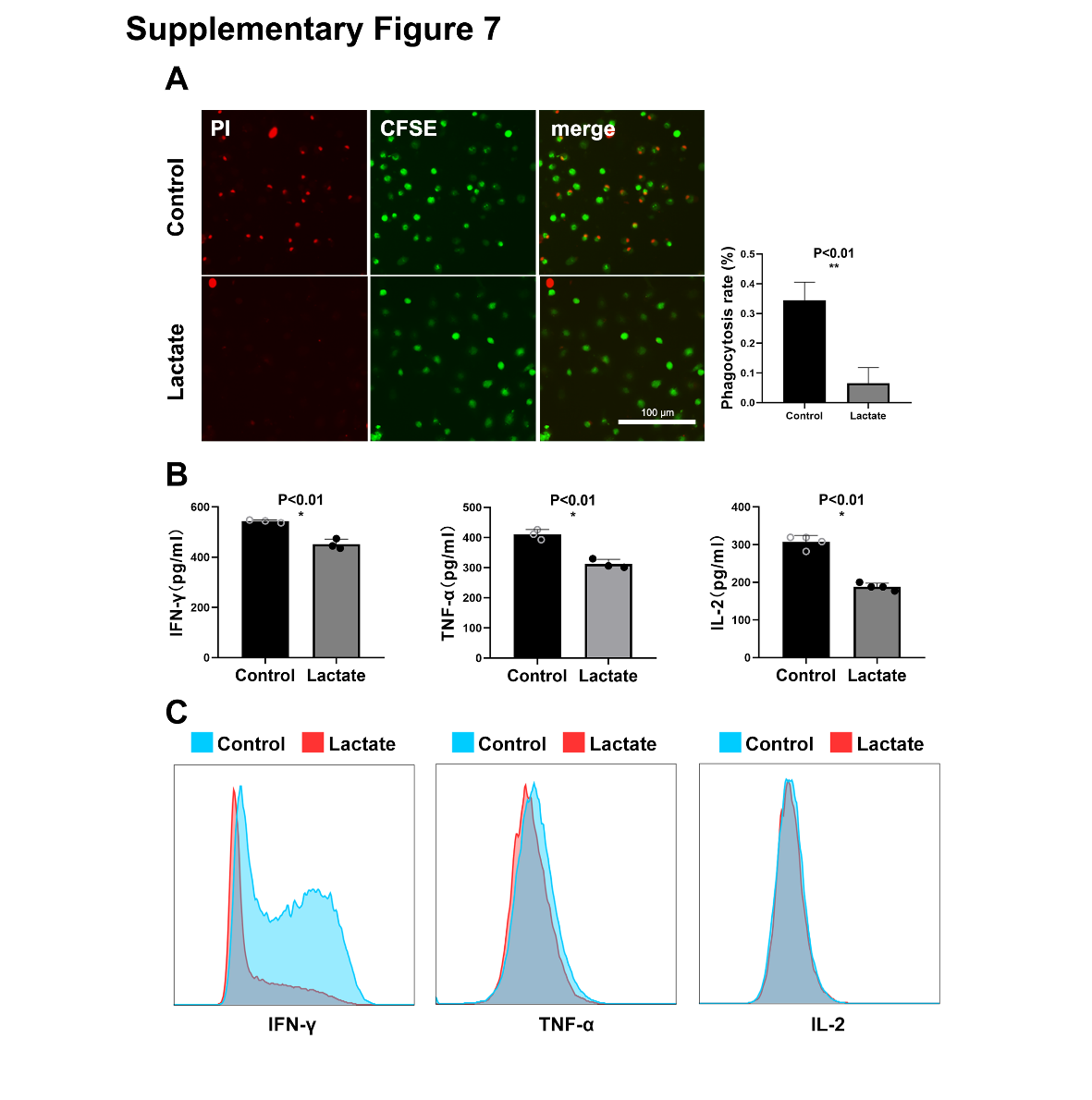


**(A)** Representative fluorescence images showing phagocytosis of apoptotic MTCQ1 cells by TAMs after lactate stimulation. Scale bar: 100 μm. **(B)** ELISA shows that lactate treatment significantly reduces IFN-γ, TNF-α, and IL-2 concentrations in macrophage–OT-II CD4⁺ T cell coculture (all P < 0. 01). **(C)** Representative images showing effects of lactate on macrophage-induced CD4⁺ T cell activation and cytokine secretion.

**Fig. S8.**

**
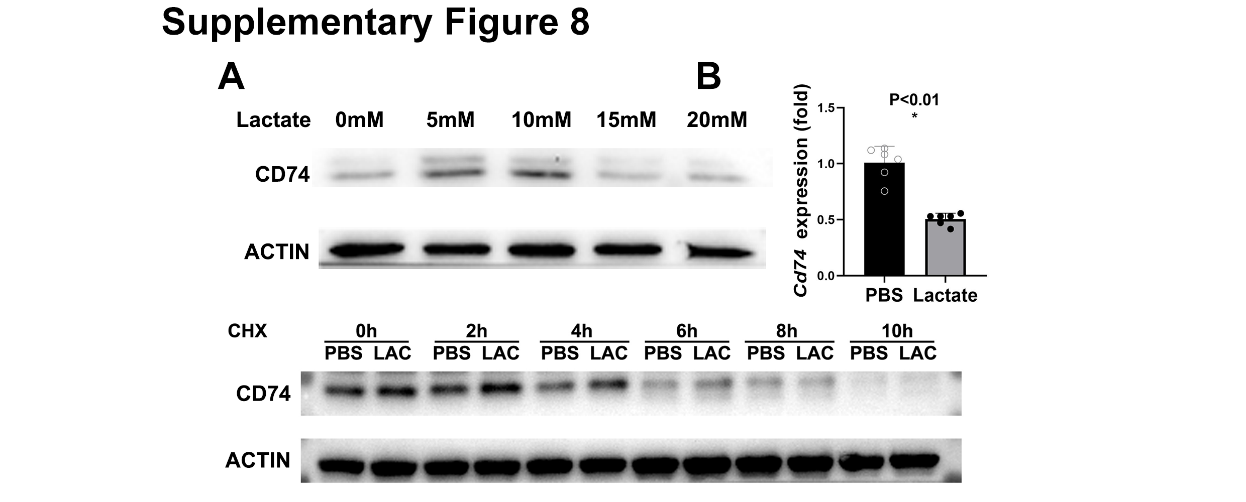
**

**(A)** Western blot showing CD74 expression in BMDMs treated with various lactate concentrations. **(B)** RT-PCR showing *Cd74* mRNA levels in BMDMs treated with 20 mM lactate. **(C)** Cycloheximide chase assay detecting CD74 protein stability at 0, 2, 4, 6, 8, and 10 hours after 20 mM lactate treatment.

**Fig. S9.**

**
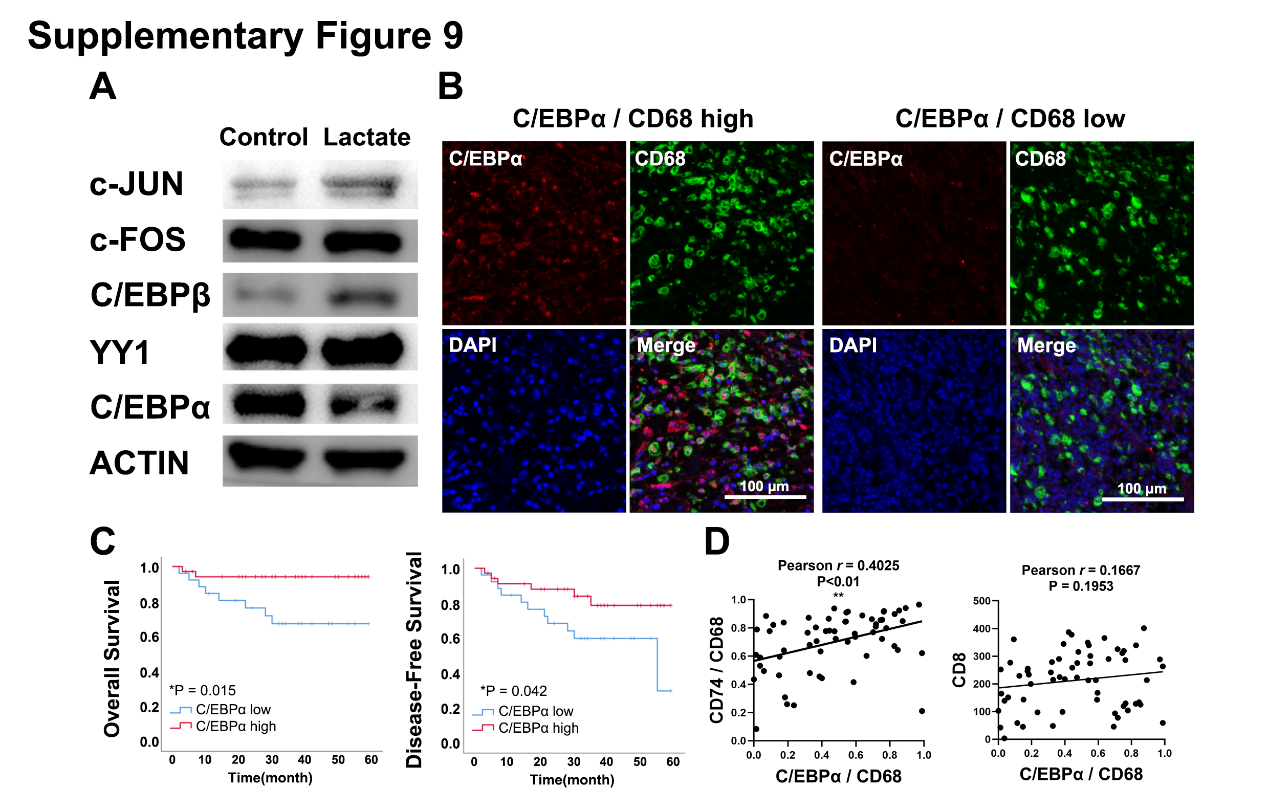
**

**(A)** Western blot shows lactate stimulation upregulates c-JUN and C/EBPβ expression, has no significant effect on c-FOS and YY1, and downregulates C/EBPα. **(B)** Representative immunofluorescence staining of C/EBPα and CD68 in OSCC patient tumor sections (n=61). Scale bar: 100 μm. **(C)** Kaplan–Meier survival analysis shows that patients in the C/EBPα-high group exhibit significantly longer overall survival and disease-free survival (P = 0.015 and P = 0.042, respectively). **(D)** Correlation analysis of C/EBPα/CD68 with CD74/CD68 (r = 0.403, P < 0.01) and CD8^+^ cells(r = 0.167, P = 0.195) in OSCC patients.

**Fig. S10.**

**
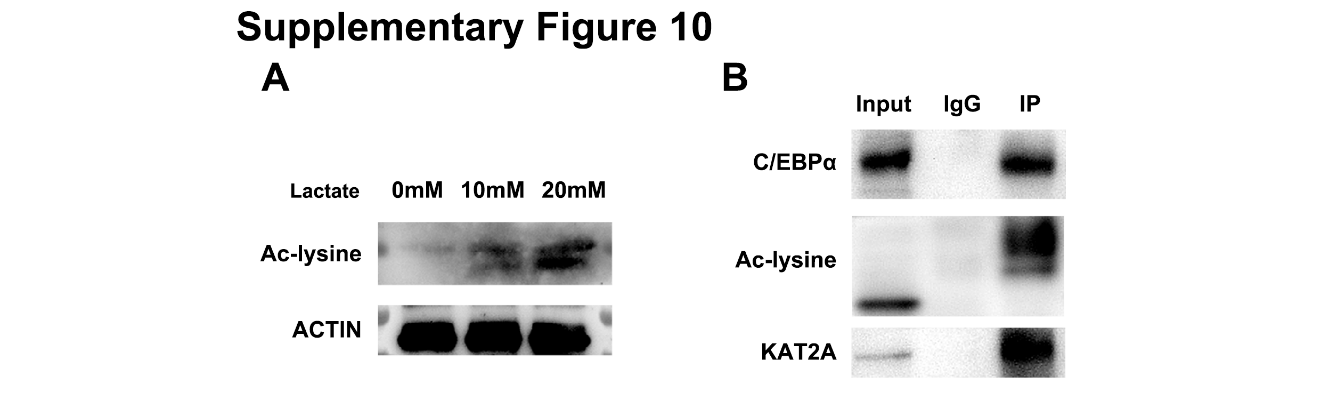
**

**(A)** Lactate activates global protein acetylation, with higher lactate levels increasing total acetylation. **(B)** Co-IP confirms that C/EBPα interacts with Acetylated-Lysine (Ac-lysine) and KAT2A after lactate treatment.

**Fig. S11.**

**
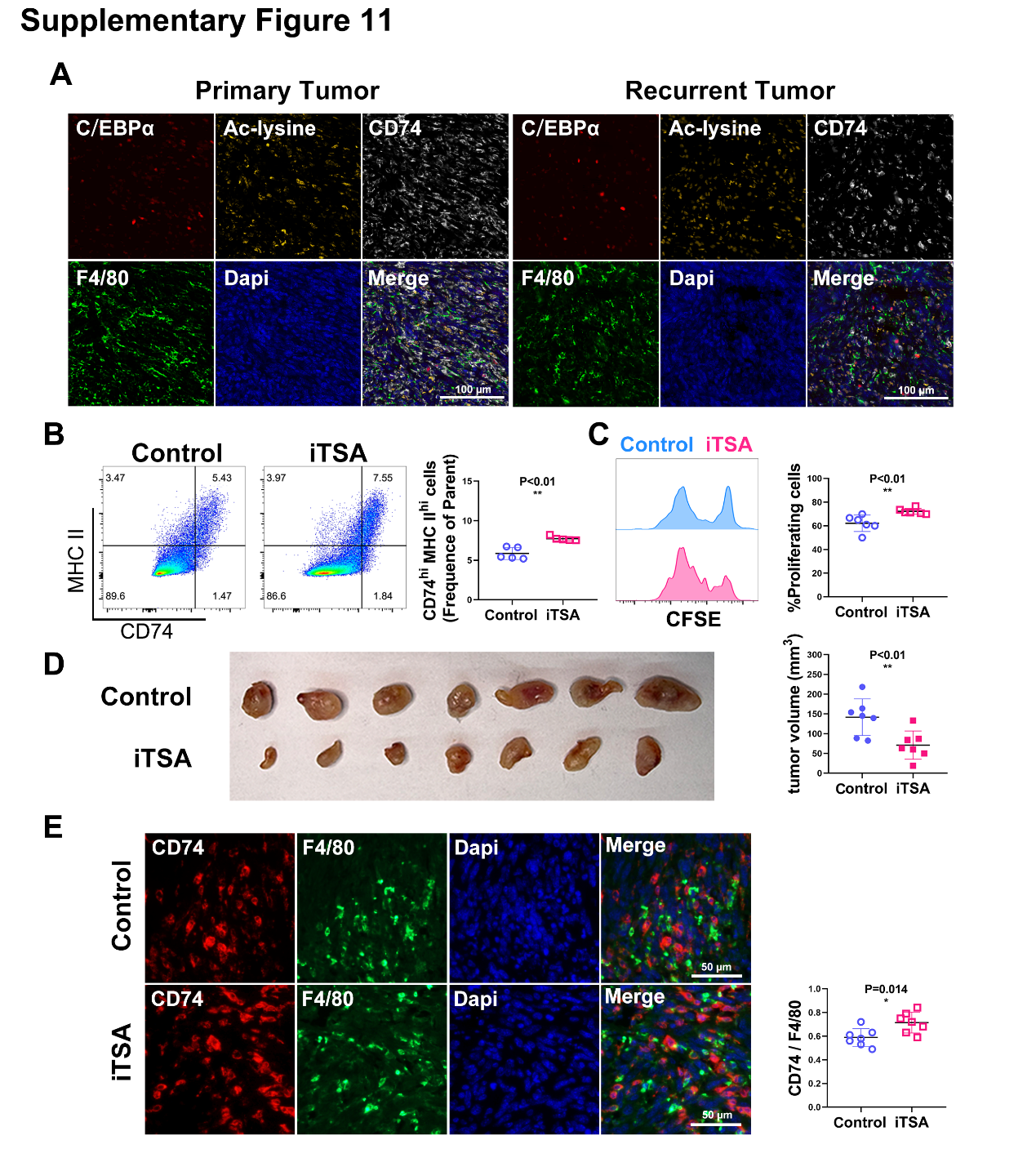
**

**(A)** mIHC staining showed that more TAMs with Ac-Lysine/C/EBPα co-localization exhibit reduced CD74 expression in recurrent tumor models. Scale bar: 100 μm. **(B)** Flow cytometry showed that treatment with the HDAC activator iTSA significantly increases the frequency of CD74^hi^ TAMs. n = 5; P < 0.01. **(C)** iTSA partially restored antigen-presentation capacity of TAMs. n = 6; P < 0.01. **(D)** In vivo iTSA administration significantly reduces tumor volume in OSCC subcutaneous tumor models. n = 7; P < 0.01. **(E)** mIHC shows increased CD74 expression in tumor-infiltrating macrophages following iTSA treatment (P = 0. 014). Scale bar: 50 μm.

**Table S1. Chi-square test of macrophage subset proportions**

| celltype | Group1 | Group2 | Chi2 | df | p.value | p.adj | Significance |
| --- | --- | --- | --- | --- | --- | --- | --- |
| CD74 MACRO | R | T | 31.85991 | 1 | 1.66E-08 | 1.66E-08 | *** |
| CD163 MACRO | R | T | 8.78E-30 | 1 | 1 | 1 | ns |
| ITGA9 MACRO | R | T | 0.876767 | 1 | 0.3490887 | 0.3490887 | ns |
| SPP1 MACRO | R | T | 36.93331 | 1 | 1.22E-09 | 1.22E-09 | *** |

**Table S2. Primer sequences for qRT-PCR**

| Primers | Sequences (5’–3’) |
| --- | --- |
| β-ACTIN-F | GGCTGTATTCCCCTCCATCG |
| β-ACTIN-R | CCAGTTGGTAACAATGCCATGT |
| C/EBPα-F | TTACAACAGGCCAGGTTTCC |
| C/EBPα-R | CTCTGGGATGGATCGATTGT |
| MCT1-F | GGTGGAGGTCCTATCAGCAGT |
| MCT1-R | CAGAAAGAAGCTGCAATCAAGC |
| MCT4-F | CCATGCTCTACGGGACAGG |
| MCT4-R | GCTTGCTGAAGTAGCGGTT |

**Table S3. Primer sequences for Chip-qPCR**

| Primers | Sequences (5’–3’) |
| --- | --- |
| CD74-C/EBPα-Site1-F | TTGTTAGGGAGCGAATAGGC |
| CD74-C/EBPα-Site1-R | GGCTGGCCTCAAACTCACTA |
| CD74-C/EBPα-Site2-F | CAATATCCTCTGGGCTGTGG |
| CD74-C/EBPα-Site2-R | GAGCAGTCGGGTGCTCTTAC |
| CD74-C/EBPα-Site3-F | TCCCAAGGCCTTAGTGATTC |
| CD74-C/EBPα-Site3-R | ATGGTTGGAGCCCAGATTTT |

**Table S4. Fluorochrome-conjugated antibodies used in flow cytometry**

| **Antibody** | **RRID** | **Supplier** |
| --- | --- | --- |
| BV605 Anti Mouse F4/80 Antibody | AB_2562305 | BD Pharmingen, USA |
| PE Anti Mouse CD3 Antibody | AB_10804049 | eBioscience, USA |
| APC Anti Mouse CD8 Antibody | AB_1569931 | eBioscience, USA |
| FITC Anti Mouse CD4 Antibody | AB_1569721 | eBioscience, USA |
| PerCP/Cyanine5.5 Anti Mouse CD11b Antibody | AB_3713129 | Biolegend, USA |
| Alexa Fluor 647 Anti mouse CD74 Antibody | AB_2632608 | Biolegend, USA |
| BV650 Anti Mouse CD69 Antibody | AB_2616934 | BD Pharmingen, USA |
| BUV395 Anti Mouse CD45 Antibody | AB_2651134 | BD Pharmingen, USA |
| BV421 Anti Mouse I A/I E Antibody | AB_2716857 | BD Pharmingen, USA |
| PE/Cyanine7 Anti Mouse CD279/PD 1 Antibody | AB_3696486 | Elabscience, China |
| PerCP/Cyanine5.5 Anti Mouse CD25 Antibody | AB_2621889 | Elabscience, China |
| BV650 Anti mouse TNFα Antibody | AB_2562450 | Biolegend, USA |
| BV421 Anti mouse IL 2 Antibody | AB_2650897 | Biolegend, USA |
| PE Cy7 Anti Mouse IFNγ Antibody | AB_3677582 | BD Pharmingen, USA |
| Alexa Fluor 488 Anti Rabbit IgG | AB_2819209 | Proteintech, China |
| Fixable Viability Dye eFluor™ 780 |  | eBioscience,USA |

**Table S5. Antibodies for mIHC**

| **Antibody** | **RRID** | **Supplier** |
| --- | --- | --- |
| Rabbit Anti Human CD8 Antibody | AB_1991073 | CST, USA |
| Rabbit Anti Human CD68 Antibody | AB_1931368 | CST, USA |
| Rabbit Anti Human CD74 Antibody | AB_10790707 | CST, USA |
| Anti Mouse F4/80 Antibody | AB_10888718 | CST, USA |
| Anti Mouse CD3 Antibody | AB_726325 | Abcam, UK |
| Anti Mouse CD4 Antibody | AB_1648505 | CST, USA |
| Anti Mouse CD74 Antibody | AB_1792435 | Abcam, UK |

**Table S6.** **Antibodies for WB and IP**

| **Antibody** | **RRID** | **Supplier** |
| --- | --- | --- |
| Anti Mouse β-ACTIN Antibody | AB_3675583 | Proteintech, USA |
| Anti Mouse iNOS Antibody | AB_1078202 | Abcam, UK |
| Anti Mouse CD206 Antibody | AB_3671113 | Proteintech, USA |
| Anti Mouse MCT1 Antibody | AB_2878645 | Proteintech, USA |
| Anti Mouse MCT4 Antibody | AB_11182479 | Proteintech, USA |
| Anti Mouse LDHA Antibody | AB_10858925 | Proteintech, USA |
| Anti Mouse C/EBPα Antibody | AB_562253 | Abcam, UK |
| Anti Mouse KAT2A Antibody | AB_2280551 | Proteintech, China |
| Anti Mouse Ac lysine Antibody | AB_1118639 | Santa Cruz, USA |
| Anti Rabbit IgG (Light Chain) | AB_2890988 | Proteintech, China |

**Table S7.** **Primer sequences and expected PCR product sizes for mouse genotyping**

| Number | Name | Sequence | Product size | Information |
| --- | --- | --- | --- | --- |
| 1 | Cd74-3wt-tF1 | GCAAAGTGCTTCTTCACCTTAGC | WT:338bp | Cd74-flox |
| GC:41% | Cd74-3wt-tR1 | CCTTCCCAATAGCAGAGTTTGC | Fl:441bp |  |
| 2 | Lyz2-KI-tF2 | ATGAAGTGCAAGAACGTGGTGC | KI:433bp | Lyz2-CreERT2 |
|  | Lyz2-KI-tR2 | TTCTGAGCGATTAGCTGGAGC | | |
| 3 | Lyz2-wt-tF1 | GCACACAGCTCAAATGTAGGAAACC | WT:496bp | |
|  | Lyz2-wt-tR1 | ATTTAGTGACAGTCCCTGAGCCC | KI:2530bp | |
